# High-energy and durable lithium metal batteries using garnet-type solid electrolytes with tailored lithium-metal compatibility

**DOI:** 10.1038/s41467-022-29531-x

**Published:** 2022-04-06

**Authors:** Sewon Kim, Ju-Sik Kim, Lincoln Miara, Yan Wang, Sung-Kyun Jung, Seong Yong Park, Zhen Song, Hyungsub Kim, Michael Badding, JaeMyung Chang, Victor Roev, Gabin Yoon, Ryounghee Kim, Jung-Hwa Kim, Kyungho Yoon, Dongmin Im, Kisuk Kang

**Affiliations:** 1 Department of Materials Science and Engineering, Seoul National University, 1 Gwanak-ro, Gwanak-gu, Seoul, 08826 Republic of Korea; 2 Battery Material Lab, Samsung Advanced Institute of Technology, Samsung-ro 130, Yeongtong-gu, Suwon-si, Gyeonggi-do, 16678 Republic of Korea; 3 Advanced Materials Lab, Samsung Advanced Institute of Technology-America, Samsung Semiconductor, Inc., Cambridge, 02138 MA USA; 4 Analytical Engineering Group, Samsung Advanced Institute of Technology, Samsung-ro 130, Yeongtong-gu, Suwon-si, Gyeonggi-do, 16678 Republic of Korea; 5 Sullivan Park Campus, Corning Incorporated, 21 Lynn Morse Rd, Painted Post, NY 14870 USA; 6 Korea Atomic Energy Research Institute, Daedeok-daero 989 Beon-Gil, Yuseong-gu, Daejon, 34507 Republic of Korea; 7 Department of Materials Science and Engineering, Research Institute of Advanced Materials (RIAM), Seoul National University, 1 Gwanak-ro, Gwanak-gu, Seoul, 08826 Republic of Korea; 8 Center for Nanoparticle Research, Institute of Basic Science, Seoul National University, 1 Gwanak-ro, Gwanak-gu, Seoul, 08826 Republic of Korea; 9 Institute of Engineering Research, College of Engineering, Seoul National University, 1 Gwanak-ro, Gwanak-gu, Seoul, 08826 Republic of Korea; 10 School of Chemical and Biological Engineering, Seoul National University, 1 Gwanak-ro, Gwanak-gu, Seoul, 08826 Republic of Korea; 11 Present Address: Department of Energy Engineering, School of Energy and Chemical Engineering, Ulsan National Institute of Science and Technology (UNIST), Ulsan, 44919 Republic of Korea

**Keywords:** Batteries, Batteries

## Abstract

Lithium metal batteries using solid electrolytes are considered to be the next-generation lithium batteries due to their enhanced energy density and safety. However, interfacial instabilities between Li-metal and solid electrolytes limit their implementation in practical batteries. Herein, Li-metal batteries using tailored garnet-type Li_7-x_La_3-a_Zr_2-b_O_12_ (LLZO) solid electrolytes is reported, which shows remarkable stability and energy density, meeting the lifespan requirements of commercial applications. We demonstrate that the compatibility between LLZO and lithium metal is crucial for long-term stability, which is accomplished by bulk dopant regulating and dopant-specific interfacial treatment using protonation/etching. An all-solid-state with 5 mAh cm^−2^ cathode delivers a cumulative capacity of over 4000 mAh cm^−2^ at 3 mA cm^−2^, which to the best of our knowledge, is the highest cycling parameter reported for Li-metal batteries with LLZOs. These findings are expected to promote the development of solid-state Li-metal batteries by highlighting the efficacy of the coupled bulk and interface doping of solid electrolytes.

## Abstract (web summary)

Lithium-metal batteries (LMBs) have attracted intense interest but the instability issues limit its practical deployment. Here, the authors report a durable LMB with high energy density using a garnet-type solid electrolyte with a tailored Li-metal compatibility.

## Introduction

One of the viable options to increase the energy densities of lithium-ion batteries (LIBs), taking full advantage of the state-of-the-art LIB technology, is to adopt Li-metal anode in the cell, which affords the highest theoretical capacity (3860 mAh g^−1^) among the anode materials^1,2^. However, practical limitations such as dendrite growth, low Coulombic efficiency, and safety issues remain unresolved despite extensive efforts to apply Li-metal anode in LIBs^3–5^. Recent progress in the development of solid-state electrolytes has provided a promising new opportunity for using Li-metal anodes, whose mechanical rigidity can effectively suppress lithium dendrite short-circuiting, which together with the non-flammable characteristics can secure the safety of a battery^6,7^. Garnet-type oxide electrolytes, e.g., Li_7_La_3_Zr_2_O_12_ (LLZO), are some of the leading candidates for Li-metal solid-state batteries, and show high ionic conductivities at room temperature (~1 mS cm^−1^), along with excellent chemical stability with lithium metal^8–10^. Owing to the stability of LLZO in ambient air, which is beneficial in the fabrication of solid-state batteries, it is widely considered a promising and feasible solid electrolyte. Nevertheless, no prior study to date has reported that the LLZO-based Li-metal batteries with acceptable electrochemical performances for practical applications. This is primarily attributed to the unexpected short-circuiting caused by lithium metal piercing through the LLZO at practical current densities^11,12^. Various mechanisms underlying this phenomenon have been proposed, including crack propagation induced by stress concentration on the LLZO surface^13–16^ and non-uniform current distribution caused by poor contact between the Li metal and LLZO^17–19^.

Recent studies of lithium penetration suggested that the electronic conductivity of LLZO can contribute to substantial lithium formation inside LLZO, causing premature short-circuiting^12,20^. Although the underlying mechanism is debatable, studies have begun to reveal that the non-zero electronic conductivity of solid electrolyte can promote lithium nucleation both in the bulk and at the grain boundary of the electrolyte^12,21–23^. In particular, presumably higher electronic conductivity along the grain boundary may facilitate nucleation and growth of the lithium metal throughout the electrolyte, expediting the short-circuiting between the two electrodes^23,24^. In addition, previous literature reports have suggested that the doped-LLZOs can yield electronically conductive by-products via chemical/electrochemical reduction upon contact with lithium metal at the interface^10,25,26^. If lithium metal precipitates along the grain boundaries, the subsequent by-products with high electronic conductivity cannot passivate the decomposition reaction and therefore would more critically accelerate the short-circuiting along the grain boundaries in the doped-LLZO solid-state electrolytes. As various dopants have been used to optimise the ionic conductivity of LLZOs^26–28^, these possibilities should not be overlooked.

In this study, we particularly pay attention to the potential by-product formation along grain boundaries by tailoring the LLZO-based solid electrolyte for stability and passivation against lithium penetration. The original idea is to adjust the by-products of LLZO by selecting suitable dopants for the grain boundary and bulk, considering the compatibility between doped-LLZO and lithium, without compromising the overall ionic conductivity. To achieve this goal, we investigate the stabilities of bulk LLZOs doped with various dopants with lithium metal. A subsequent process to selectively alter the grain boundaries by solution-based etching that permeates LLZO solid-electrolyte pellets is implemented. It is envisioned that such treatment would accompany a substantial compositional change specifically at the grain boundary, e.g., lithium substitution by protons during acid etching^29,30^. This is expected to suppress the formation of conductive by-products, while preserving the high bulk ionic conductivity of doped-LLZO. Besides, we reveal our interfacial treatment method effectively releases the residual stress in LLZO and aid in the maintenance of an intact contact at the interface.

In the following discussion, it is demonstrated that the rational selection of dopants and etching agents can lead to a significant enhancement in the performance of a practical full cell consisting of a conventional cathode (i.e., LiNi_1/3_Co_1/3_Mn_1/3_O_2_ (NCM111)) at a commercially applicable loading capacity with a thin lithium metal anode (20 µm) and LLZO electrolyte. The cell delivers an areal capacity of 3.2 mAh cm^−2^ for 1000 cycles at C/2 rate with a capacity retention of 95.0%. The feasibility of the new cell with a 110-μm-thick Ta–LLZO solid electrolyte further indicates its potential in delivering a remarkable energy density of 470 Wh L^−1^. More importantly, an all-solid-state cell using a 5-mAh cm^−2^ composite cathode also delivers 4000 mAh cm^−2^ at 3 mA cm^−2^. This is the first report that describes solid-state cells with a Li-metal anode that can meet the lifespan requirements of general commercial applications: (i) 500 cycles without Li-metal shorting; (ii) at an operating current density of 1.5 mA cm^−2^ (0.5 C rate); (iii) with Li-metal utilisation per cycle of over 3 mAh cm^−2^ ^31,32^. These findings are expected to advance the development of solid-state batteries with garnet solid electrolytes by highlighting that a coupled approach to designing the bulk and grain boundaries of the solid electrolyte plays a key role in achieving the long-term stability of solid-state batteries.

## Results and discussion

### Chemical stabilities of doped-LLZOs against Li metal

**Fig. 1:**
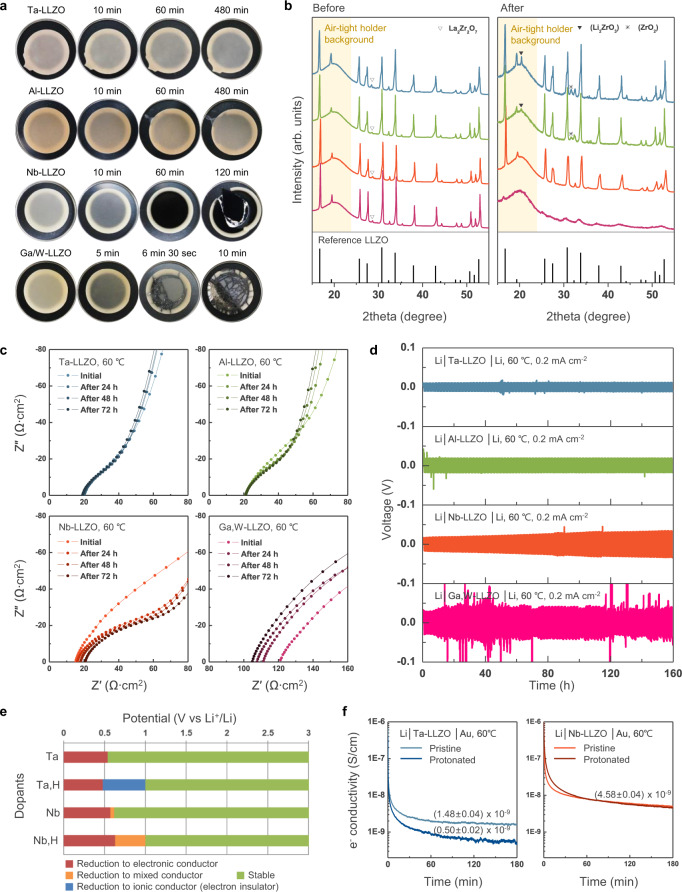
Dopant stability of LLZO against lithium metal. **a** Optical images of LLZO pellets in contact with Li metal at 200 °C over time after assembly under a cold-isostatic pressure of 250 MPa. **b** Comparison of XRD patterns of LLZO pellets with reference XRD pattern (ICSD 01-080-6142) (left) before and (right) after contact with lithium metal. **c** Evolution of the electrochemical impedance spectra over time for the Li/LLZO/Au cells measured at 60 °C. **d** Galvanostatic cycling of Li/LLZO/Li symmetric cells at 60 °C with a 0.2 mA cm^–2^ current density. **e** Electrochemical stability windows for pristine and protonated Ta– and Nb–LLZOs calculated using the DFT. **f** Electronic conductivities of Ta– and Nb–LLZO pellets before and after protonation measured by DC polarisation with an applied voltage of 0.5 V at 60 °C.

Doping the LLZOs is important to attain a high ionic conductivity of the LLZO solid electrolyte^33–35^; however, the stability of doped LLZO with Li metal may substantially differ depending on the dopants^28,36–38^. To compare the effect of the dopant on the stability, chemical colouration^39^ tests were performed, which visually showed the reactivity between the lithium metal and LLZO pellets (Fig. 1a; see Methods section for details). The test was performed using LLZOs with four representative dopants: Li_6.5_La_3_Zr_1.5_Ta_0.5_O_12_ (Ta–LLZO), Li_6.25_Al_0.25_La_3_Zr_2_O_12_ (Al–LLZO), Li_6.5_La_3_Zr_1.5_Nb_0.5_O_12_ (Nb–LLZO) and Li_4.9_Ga_0.5_La_3_Zr_1.7_W_0.3_O_12_ (Ga,W–LLZO); these dopants have been most commonly used to increase the Li-ion conductivity or density of the LLZO pellet^33–35^. The physical properties of doped LLZO electrolyte pellets such as their crystal structures, morphology with relative density, and ionic conductivity are presented in the supplementary information (Supplementary Figs. 1–4). To accelerate the potential reaction between Li metal and LLZO, the pellets were heated to 200 °C, which was close to the melting point of Li metal, over the observation period. Figure 1a shows the difference in the stabilities of the LLZOs against Li metal depending on the dopants. Initially, the Li was plainly visible beneath the thin LLZO pellet for all the samples without an apparent side reaction. In the case of Ta– and Al–LLZOs, the contact area slightly darkened over time; however, no significant change in colour was observed even after 8 h. Meanwhile, Nb– and Ga,W–LLZOs showed pronounced degradation under the same experimental conditions. The contact area of Nb–LLZO began to darken from the contact area within only 10 min, subsequently becoming black and fracturing into pieces after 1 h. This observation was consistent with those reported in prior studies, where Nb-containing garnet-type solid electrolytes discoloured after the electrochemical test, suggesting that Nb^5+^ could have been reduced to Nb^4+^^38,40^. Ga,W–LLZO showed more severe changes in a few minutes. In addition to the colour change, the Li metal penetrated the pellet (Supplementary Fig. 5). The reaction proceeded until the Li was completely consumed, and the entire pellet turned black with significant pulverisation. Considering that LLZOs with four different compositions did not show noticeable differences in the crystal structures, relative phase amounts, and microstructures, but only differed in the dopant characteristics, the rapid colour change and/or Li metal penetration in these experiments suggested that the LLZO with certain dopants could substantially react with Li during long-term cycling of the batteries.

The doped-LLZO samples were carefully analysed after the colouration tests (Supplementary Figs. 6–9). Energy-dispersive X-ray spectroscopy (EDS) data (Supplementary Fig. 6) revealed a significant segregation of La and Zr with the extensive micro-crack formation in the case of Nb– and Ga,W–LLZO, indicating instability upon prolonged exposure to lithium metal, while Ta–and Al–LLZO remained intact. The cross-sectional lithium distribution examined by secondary ion mass spectrometry (Supplementary Fig. 7) also indicated that the entire Nb– and Ga,W–LLZO pellets reacted with lithium metal blurring the initial boundary between the Li and LLZO, while Ta–LLZO was less affected. Consistent with the chemical colouration results, the X-ray diffraction (XRD) patterns (Fig. 1b) showed substantially broadened peaks for Ga,W–LLZO, indicating the loss of crystallinity owing to the side reaction with lithium metal, accompanied by reduction of the electrolyte (Supplementary Fig. 8) and pellet pulverisation. The peaks for Nb–LLZO were also altered due to the changes of the lattice parameters and the relative phase amounts of the cubic and tetragonal phases (Supplementary Fig. 9), accompanied by Li insertion into the garnet structure with Nb^5+^ reduction^41^. Notably, when the cross-section of the reacted Nb–LLZO pellet was examined, Li had propagated through the grain boundary region^42^ (Supplementary Fig. 10). This indicated that, even without electrochemical bias, the chemical instability of doped-LLZO against Li could lead to high Li metal penetration through the solid-state electrolyte along the grain boundaries. In addition, Ta– and Al–LLZOs, which appeared stable during the chemical colouration test, partially reacted with Li, resulting in new sets of XRD peaks at approximately 20.4° and 31.6°. These peaks could be assigned to Li_2_ZrO_3_ and ZrO_2_, respectively, implying that Ta- and Al–LLZOs underwent side reactions even though they appeared stable at the macroscale, which presumably occurred locally, unlike in the case of Nb– and Ga,W–LLZO.

The observed chemical stability of the doped LLZOs was proportionally correlated with the electrochemical performance of Li/LLZO solid-state cells. Figure 1c shows the electrochemical impedance spectroscopy (EIS) results for Li/doped-LLZO/Au half-cells as a function of time (see Supplementary Note 1 for details). The EIS spectra of Ta– and Al–LLZO showed minor changes in the low-frequency semi-circle tail over time, indicating a slight increase in the interfacial impedance. In contrast, the spectra of Nb– and Ga,W–LLZOs were significantly altered with respect to both the low-frequency interfacial impedance and high-frequency region corresponding to the resistance of the bulk LLZO electrolyte. Nb–LLZO showed an additional interfacial impedance, which can be attributed to the substantial formation of by-products at the interface^40^, together with an increase in the bulk resistance due to the degradation of the garnet structure^43^. (See Supplementary Fig. 11 for more details.) For Ga,W–LLZO, the impedance decreased over time; however, this was due to substantial lithium propagation into the pellet, which decreased the geometric distance between the electrodes. The relative stability of the EIS spectra for Ta- and Al-doped LLZOs compared to those of Nb- and Ga,W-doped LLZOs is consistent with the tendency observed through the chemical reaction with lithium metal. This is also critical in the galvanostatic cycling performances of Li/LLZO/Li symmetric cells. Figure 1d shows the time-dependent voltage profile of each symmetric cell for electrochemical lithium deposition/stripping at a current density of 0.2 mA cm^−2^ at 60 °C. The cells with Ta– or Al–LLZO showed relatively stable voltage profiles during 160 h of cycling. In contrast, the cell with Nb–LLZO showed a continuous increase in polarisation over repeated cycles, which is in agreement with the EIS result showing increasing bulk and interfacial resistances over time. Comparing the EIS results before and after cycling, both the bulk and interfacial resistances changed significantly (Supplementary Fig. 12). Ga,W–LLZO showed an unstable profile with large perturbations in voltage, suggesting that dynamic short circuits were formed inside the solid electrolyte^44,45^.

To elucidate the observed distinct stabilities of the LLZOs, even with small dopant concentrations, theoretical calculations to determine the thermodynamic stability of the doped-LLZOs were performed using the density functional theory (DFT). Grand potential phase diagrams were constructed^46,47^, and the stabilities of the doped-LLZOs were examined against a reservoir of lithium (Supplementary Note 2 and Supplementary Fig. 13). These results show that LLZOs become highly susceptible to reduction by lithium metal in the presence of dopants, yielding metallic by-products, which is consistent with previous reports describing that very few metal dopants in oxide systems are stable against lithium^26,27^. All dopants, including Ta and Al, tend to diminish the reduction stability of the LLZOs compared to that of the undoped LLZO in the order of Ga,W–, Nb–, Ta–, and Al. Upon exposure to lithium, the reduction of the LLZOs leads to the formation of electronically conductive by-products such as Ta, Al_3_Zr, W/Ga, LiNbO_2_ (followed by complete reduction to Nb), and Zr_3_O, as indicated by the red and orange bars in Supplementary Fig. 13. The presence of metallic by-products can lead to a failure in passivating the reaction between lithium metal and doped-LLZO, rapidly propagating the reductive decomposition throughout the LLZO electrolyte.

### Selective passivation of doped-LLZOs along the grain boundaries and interface

As chemical/electrochemical reduction by lithium is rarely avoidable, even for relatively stable dopants such as Ta or Al, attempts to further tailor the compositions of the doped LLZOs with secondary dopants were made such that the by-products from any decomposition were electronically insulating but ionically conducting. Moreover, to minimise the effect of secondary doping on the overall ionic conductivities of LLZOs, the compositional tailoring was limited to the interface of LLZO, including the grain boundaries. Considering the practical feasibility of the selective doping of the interface, a possible solution-based process was considered, which could permeate through the grain boundaries of the LLZOs. In addition, inspired by the conventional etching technique to visualise the grain boundaries of pellet samples by scanning electron microscopy (SEM)^48^, the acid etching process was employed to exclusively alter the grain boundaries and interface. It was speculated that acid etching could be a feasible method to dope protons, because the ion exchange between lithium and proton could occur when LLZO is immersed in an acid solution, which could also be reversed using LiNO_3_-containing solutions^49^. Therefore, the degree of protonation could be empirically modified to balance the potential increase in stability with the expected decrease in ionic conductivity. The DFT thermodynamic assessment supported the hypothesis that exchanging lithium with a proton in the LLZOs aided the production of passivating by-products during reduction at the Li/LLZO interface (Supplementary Tables 2 and 3). The calculations predicted that insulating by-products such as hydroxides (La(OH)_3_ and LiOH) could form during the reduction of Ta- and Al-doped systems, which could passivate further decomposition. Interestingly, as shown in Fig. 1e and Supplementary Table 3, the co-doping of Ta and proton in LLZO leads to the formation of the insulating Li_3_TaO_4_ phase in addition to hydroxides at approximately 1.0 V (vs. Li/Li^+^), while the doping of only Ta in LLZO yields a metallic Ta phase as the by-product. This implies that when reductive decomposition is initiated for the proton-doped Ta–LLZO, the reaction route would first produce these electronically insulating (and ionically conducting) phases that passivate further reduction to metallic phases. A similar phenomenon was observed for the co-doping of Nb and proton, which produced Li_8_Nb_2_O_9_ as an intermediate by-product during the decomposition reaction. However, the Li_8_Nb_2_O_9_ phase is considered a mixed electronic ionic conductor due to its small band gap energy^50^, and is therefore expected to be unsuccessful in passivating the decomposition. The difference between Ta and Nb doping indicates that the secondary dopant should be rationally selected specifically considering the primary dopant of LLZO.

Inspired by these results, we protonated the grain boundaries and interfaces of doped LLZOs by a secondary doping process via the acid treatment of 350-µm doped-LLZO pellets. The protonation of the samples was confirmed by thermal gravimetric analysis performed under N_2_ atmosphere (Supplementary Fig. 14). An apparent weight loss was observed for the protonated LLZO pellet at approximately 450 °C, which was attributed to the release of hydrogen from the pellet, whereas pristine LLZO did not show any change up to 1000 °C^30^. For the co-doped LLZOs, the stability against Li metal was comparatively evaluated (Supplementary Fig. 15). Protonated Ta– and Al–LLZO showed higher stability with lithium metal than the pristine Ta– and Al–LLZO pellets, whereas protonated Nb– and Ga,W–LLZO pellets still showed significant degradation. These results were consistent with those of the DFT calculations, which indicated the production of insulating by-products only for Ta- and Al-doped cases. The chemical colouration test also verified that protonated Ta– and Al–LLZO were stable without apparent degradation over time. Notably, the additional XRD peaks observed for pristine Ta– and Al–LLZO were not observed for their protonated counterparts, confirming the enhanced stability against lithium metal. In contrast, Nb– and Ga,W–LLZO remained unstable even after protonation, showing pellet fracture and pulverisation, which indicated the inefficacy of secondary proton doping. Nevertheless, a slight enhancement was observed for protonated Nb– and Ga,W–LLZO via EIS and cell cycling, which showed smaller impedance/polarisation at the initial cycles. Although degradation was observed in later cycles, the enhancement shown at the initial cycles was attributed to the improved interface morphology between the lithium metal and LLZOs^51^ (discussed later in detail). Figure 1f shows a comparison of the electronic conductivity of Ta– and Nb–LLZO pellets in an Au/LLZO/Li cell before and after protonation, as measured by the direct-current polarisation using the Hebb–Wagner method^52,53^ at 60 °C. Substantial electronic conductivity was measured for both pristine Ta– and Nb–LLZO pellets, probably due to the presence of conducting by-products. However, the electronic conductivity of the protonated Ta–LLZO decreases from 1.5 × 10^−9^ to 5 × 10^−10^ S cm^−1^, whereas Nb–LLZO did not show any significant change after protonation. This suggests that the suppression of the metallic by-products through the protonation of Ta–LLZO can retard the formation of electron-conducting paths in the LLZO pellet in contact with lithium metal.

### Additional effects of acid treatment on interfacial stabilisation

**Fig. 2:**
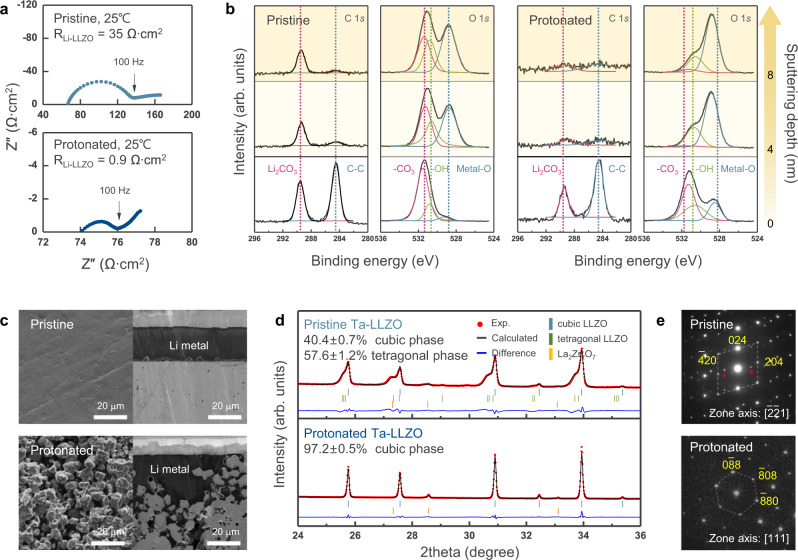
Surface stabilisation effects on Ta-doped-LLZO through acid treatment. **a** Decrease in the interfacial resistance in Li/Ta–LLZO/Li symmetric cell before and after surface tailoring. **b** XPS spectra of C 1*s* and O 1*s* region along the sputtering depth, showing the elimination of Li_2_CO_3_ below the surface. **c** SEM images of the polished and tailored pellet surfaces and cross-sections in contact with lithium metal. The protonated pellet shows pronounced rough and porous surface morphology. **d** XRD and **e** SAD patterns of Ta–LLZO before and after surface stabilisation. XRD patterns in **d** indicate that pristine Ta–LLZO includes 40.4 ± 0.7 wt% of cubic phase (${Ia}\bar{3}d$, *a* = 12.93 Å) and 57.6 ± 1.2 wt% of the tetragonal phase ($I{4}_{1}/{acd}$, *a* = 13.03 Å, *c* = 12.94 Å, *c*/*a* = 0.9928), whereas protonated Ta–LLZO consists of 97.2 ± 0.5 wt% of cubic phase (${Ia}\bar{3}d$) with a lattice parameter of 12.93 Å. The peak of the pristine pellet is clearly asymmetric due to the presence of the tetragonal phase. (Details of the Rietveld refinement are provided in Supplementary Figs. 18 and 21, and details of the refinement approaches are described in the Methods section.) In **e**, SAD pattern from the surface region of the pristine Ta–LLZO along the [$\bar{2}\bar{2}1$] zone-axis shows the spots corresponding to the tetragonal phase. The extra spots indicated by the red arrows represent the existence of an additional phase with a similar crystal structure that might be attributed to the strain field, causing double diffraction. In contrast, protonated Ta–LLZO clearly shows a diffraction pattern corresponding to a single cubic phase.

After demonstrating that doped-LLZO can be chemically stabilised by protonation, attempts to understand the possible effects of surface tailoring via acid treatment were made, and protonated Ta–LLZO was carefully investigated. Previous studies reported that acid treatment could remove the Li_2_CO_3_ layer on the LLZO surface and decrease the interfacial resistance^19,51^. In accordance, the interfacial resistance noticeably decreased from 35 Ω∙cm^2^ for the pristine pellet to 0.9 Ω∙cm^2^ for that subjected to acid treatment at 25 °C (Fig. 2a). A decrease in the interfacial resistance was also consistently observed for Al–LLZO (Supplementary Fig. 16). In addition, X-ray photoelectron spectroscopy (XPS) data (Fig. 2b) confirmed that the residual Li_2_CO_3_ on the Ta–LLZO surface was substantially reduced after protonation. The characteristic peaks of Li_2_CO_3_ at 289.5 and 531.5 eV in the C 1*s* and the O 1*s* spectra, respectively, (red dotted line in Fig. 2b) indicated that a significant amount of Li_2_CO_3_ was present on the surface of pristine LLZO, which was detected even with prolonged XPS depth profiling. In contrast, Li_2_CO_3_ peaks were observed only at the outermost surface of the protonated pellet (less than a few nanometres thick), supporting that acid treatment successfully removes the surface Li_2_CO_3_ layer. Figure 2c shows a comparison of the SEM images of the pristine and protonated Ta–LLZO pellets assembled with lithium metal. The protonated Ta–LLZO pellet was rough and porous at the surface, owing to the corrosion along the grain boundaries, in contrast to the flat surface of the pristine pellet containing only scratches. As the presence of locally distributed defects with narrow and deep profiles (such as scratches) on the electrolyte surface can initiate the Li propagation through the electrolyte and cause short-circuiting^13,15,54^, the removal of local flaws and formation of a uniform rough surface by appropriate etching is thought to mitigate lithium penetration and improve cell performance. In addition, it indicates that the reduction of the interfacial resistance is partly attributable to the increase in the effective contact area. The increase in the effective area between LLZO and Li is expected to decrease the actual areal current density^55^, contributing to a reduction in interfacial impedance, as observed for all protonated LLZO pellets (Supplementary Fig. 15).

Interestingly, during the handling of LLZO pellets in the experiments, it was observed that the mechanical properties could also be significantly enhanced by protonation. The ring-on-ring test, which is commonly used to measure the ceramic strength^56,57^, revealed that the tensile strength of the LLZO pellet increased from 86 ± 14 to 134 ± 7 MPa after acid treatment (Supplementary Fig. 17). While the origin of this strengthening is not clear, it is speculated that it is partly due to the strain release that accompanies acid treatment, which effectively removes the secondary garnet phase in the pellet (Supplementary Note 3 for further discussions). The careful analysis of Fig. 2d and Supplementary Fig. 18 shows that the pristine Ta–LLZO pellet contained a tetragonal secondary phase, accounting for ~58% based on the refinement^58,59^, as indicated by the pronounced asymmetry of the XRD peaks. Although the relative amount of the tetragonal phase is higher than that of the cubic phase, this represents a surface-limited property rather than a bulk property, as the interaction volume for XRD is near the pellet surface, owing to the limited penetration depth of X-rays in ceramics containing transition metals^60,61^. As shown in Supplementary Figs. 19 and 20, the tetragonal phase is barely detected in the powder from the crushed/ground pellet (considered representative of the overall properties), confirming that the coexistence of tetragonal and cubic phase LLZO was limited to near the surface. It has been previously reported that a significant amount of tetragonal phase can be formed when a material is insufficiently doped to stabilise the cubic polymorph^41^ or during sintering with applied pressure, and the existence of the secondary phase can result in lattice strain in the garnet structure^42^. The mechanical process including sintering, cutting, polishing, etc., to fabricate the electrolyte pellets can also induce the strain field on the surface^62^. Notably, this asymmetry was not observed after surface tailoring, indicating the removal of the secondary phase, which is consistent with the refinement results shown in Supplementary Fig. 21. It has not been confirmed if acid treatment chemically etches the secondary phase^62^ and/or the phase transformation occurs from the tetragonal to cubic phase due to lithium redistribution during the protonation process^63–65^, which warrants further investigation. Nevertheless, it was confirmed that the lattice strain in the pellet was significantly reduced by the removal of the tetragonal phase. Williamson–Hall analysis and size–strain plots^66^ in Supplementary Fig. 22 revealed that the lattice strain on the surface decreased from 0.509%, owing to the presence of the tetragonal phase in the pristine pellet, to 0.076 % in the protonated pellet. Transmission electron microscopy (TEM) analysis further confirmed the removal of the tetragonal phase and the corresponding mechanical strain. The selected-area diffraction (SAD) pattern and TEM image of the surface of the pristine pellet in Fig. 2e and Supplementary Fig. 23 clearly show that the top surface grain was composed of the tetragonal phase with a strain field, observed as fringes near the surface and double diffraction (indicated by red arrows) in the SAD pattern. In contrast, only a single pattern of the cubic phase was identified for the protonated pellet, which is in good agreement with the XRD results. Notably, the residual lattice strain in ceramics can serve as a critical driving force for crack propagation under external pressure^67^. To date, the need to enhance the mechanical properties of inorganic solid electrolytes has been relatively overlooked because the fracture toughness of most inorganic solid electrolytes is significantly higher than the elastic modulus of lithium metal. However, in the presence of significant strain in the LLZO pellets, it is speculated that the accumulated stress induced by lithium metal filling in the LLZOs can more easily trigger crack propagation, leading to the mechanical failure of LLZO electrolytes^15^.

### Electrochemical performance of solid-state Li full cell with protonated Ta*–*LLZO

**Fig. 3:**
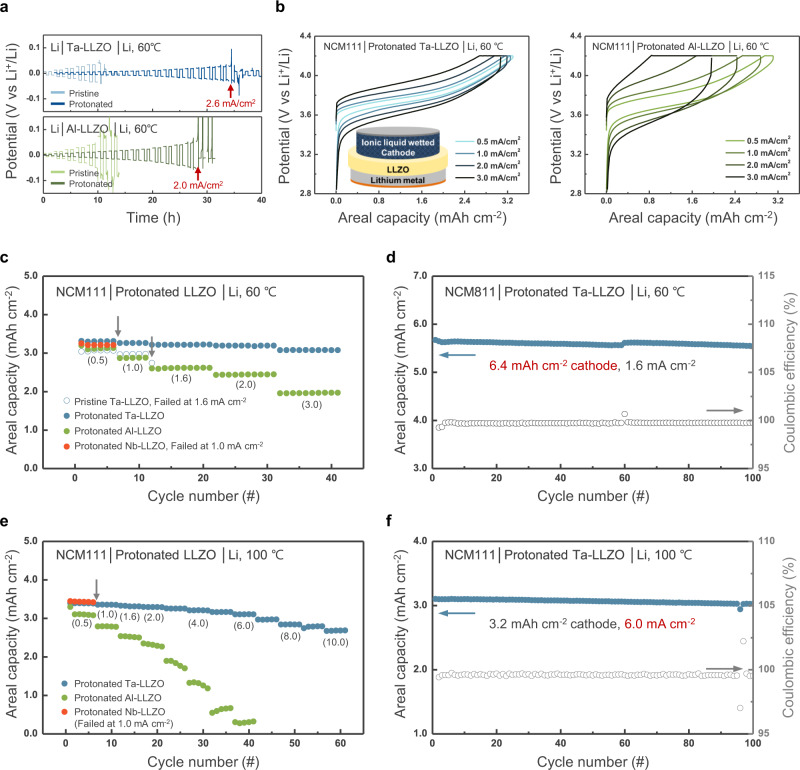
Electrochemical performance of the cells with surface-tailored LLZOs and Li-metal anodes. **a** Critical current densities of pristine (light blue and green lines) and surface-tailored (dark blue and green lines) Ta– or Al–LLZO as determined by galvanostatic cycle tests on the symmetrical cells with increasing current densities ranging from 0.1 to 1 mA cm^–2^ at a step size of 0.1 mA cm^–2^ and from 1.0 to 3.0 mA cm^–2^ at a step size of 0.2 mA cm^–2^. At each current density, the cells were cycled twice with 30 min of Li plating/stripping. **b** Voltage profiles of Li/LLZO/NCM111 cells at 60 °C with doped, surface-tailored electrolytes. **c** Rate capability for various dopants. **d** Cyclability of surface-tailored Ta–LLZO and an NCM811 cathode with a high capacity of 6.4 mAh cm^–2^ at 60 °C. A capacity of 6.0 mAh cm^–2^ corresponds to a Li-metal thickness above 30 μm. **e** Rate capability of various dopants. **f** Cyclability of surface-stabilised Ta–LLZO at 100 °C. For the rate capability test at 100 °C, the cells were operated with increasing current densities (indicated with numbers in parentheses).

To validate the effect of the enhanced stability of the tailored LLZO electrolyte on electrochemical performance, comparative electrochemical tests in lithium cell were performed (Fig. 3). Figure 3a and Supplementary Fig. 24 show the critical current densities measured for symmetric Li/LLZO/Li cells at 60 °C and 25 °C, respectively, with Ta–LLZO or Al–LLZO solid electrolytes. The critical current density represents the highest current density before the apparent short-circuiting of a symmetric cell^68,69^, and thus, it is widely accepted as a valid criterion for determining the reliability of a solid-electrolyte–Li-metal system. The figures show that the critical current density of lithium symmetric cells can be significantly improved by employing the co-doped LLZO solid electrolytes. The cells with protonated Ta– and Al–LLZO delivered critical current densities of approximately 2.6 and 2.0 mA cm^−2^, respectively, at 60 °C, and 1.6 mA cm^−2^ at 25 °C, which are remarkably higher than those obtained for the pristine equivalents. Inspired by the considerably improved stability, the tailored LLZO solid electrolyte/Li-metal anode was employed in a hybrid solid-state battery using a conventional NCM111 (LiNi_1/3_Co_1/3_Mn_1/3_O_2_) cathode with a loading capacity of 3.2 mAh cm^−2^, which corresponded to the cathode requirements for the commercial high-energy-density Li-ion cells^70^. To ensure stable contact between the thick cathode and solid electrolyte, the cathode side was slightly wetted with an ionic liquid (i.e., 2 M lithium bis(fluorosulfonyl)imide (LiFSI) in N-methyl-N-propyl pyrrolidinium bis(fluorosulfonyl)imide (Pyr13FSI)) in a hybrid solid-state battery. Figure 3b shows the charge–discharge curves and rate capability of the hybrid full cells measured at 60 °C, the temperature at which the batteries for electric vehicles are commonly tested^71,72^. The electrochemical profiles were obtained by increasing the current density stepwise; i.e., the cells were charged/discharged with current densities of 0.3 mA cm^−2^ for the first cycle, 0.5 mA cm^−2^ for the next five cycles, 1 mA cm^−2^ for the subsequent five cycles, 1.6 mA cm^−2^ for the 12th to the 21st cycle, 2 mA cm^−2^ for the 22nd to the 31st cycle, and 3 mA cm^−2^ for the 32nd to the 41st cycle. The cells delivered characteristic charge–discharge curves of the NCM111 cathode, which were sustained without any short-circuit signals (i.e., voltage noise and/or sudden drop), even at a high current density of 3 mA cm^−2^. Comparing these results with those for the cells composed of the pristine doped-LLZO electrolytes in Supplementary Fig. 25 strongly supports the hypothesis that lithium penetration in the protonated Ta– and Al–LLZO electrolytes is significantly suppressed, which allows operation at a practically high current density. In contrast, the cells with protonated Nb– and Ga,W–LLZO electrolytes short circuited at current densities below 1 mA cm^−2^, while the typical charge–discharge behaviour of the NCM111 was observed only at low current density (Supplementary Fig. 26). This result was consistent with our findings that the secondary doping of proton in Nb– and Ga,W–LLZOs did not yield insulating by-products, and thus, could not passivate the interface.

Figure 3c exemplifies that the protonated LLZO cells are capable of delivering a high rate capability without significant degradation. Approximately 93% and 63% of the capacity relative to the initial capacity at 0.5 mA cm^−2^ was delivered at 3 mA cm^−2^ for the protonated Ta–LLZO and Al–LLZO cells, respectively. Moreover, we fabricated a higher capacity cathode designed for 6.4 mAh cm^−2^, using high-nickel NCM811 (LiNi_0.8_Co_0.1_Mn_0.1_O_2_) and cycled the cell at 1.6 mA cm^−2^ (Fig. 3d and Supplementary Fig. 27). It is noteworthy that the cell with the high-capacity cathode in a protonated Ta–LLZO electrolyte provided 87% of the capacity at 1.6 mA cm^−2^ with 99.7% Coulombic efficiency after 100 cycles. The high capacity of this cathode also implies that lithium metal as thick as 32 μm could be reversibly deposited and stripped through the tailored Ta–LLZO solid electrolyte over 100 times without any notable degradation. The cells were also tested under harsher conditions, such as a high temperature of 100 °C (Fig. 3e, f). The limiting current density could further increase over 10 mA cm^−2^ for the cell with protonated Ta–LLZO, which could not be attained for the protonated Al– or Nb– LLZO system. The cell was also successfully cycled over 100 times delivering 96% of the nominal capacity (i.e., 3.2 mAh cm^−2^) at a current density of 6 mA cm^−2^ with a Coulombic efficiency of 99.6%. This indicates that the cell can be stably operated at 100 °C without short-circuiting, a temperature at which most LIBs using conventional liquid electrolytes are irreversibly damaged^71^. The cell using protonated Nb–LLZO failed to cycle at 100 °C owing to short circuits, even when operated at a low current density. This is attributed to the accelerated reaction of Nb–LLZO with Li at a higher temperature, leading to premature short-circuiting. Although the cell with protonated Al–LLZO did not show any short-circuiting, its capacity notably decreased at high current densities, showing an inferior rate capability than the case of protonated Ta–LLZO cell. Further details regarding the rate capabilities are discussed in Supplementary Figs. 28 and 29 and Supplementary Table 4.

**Fig. 4:**
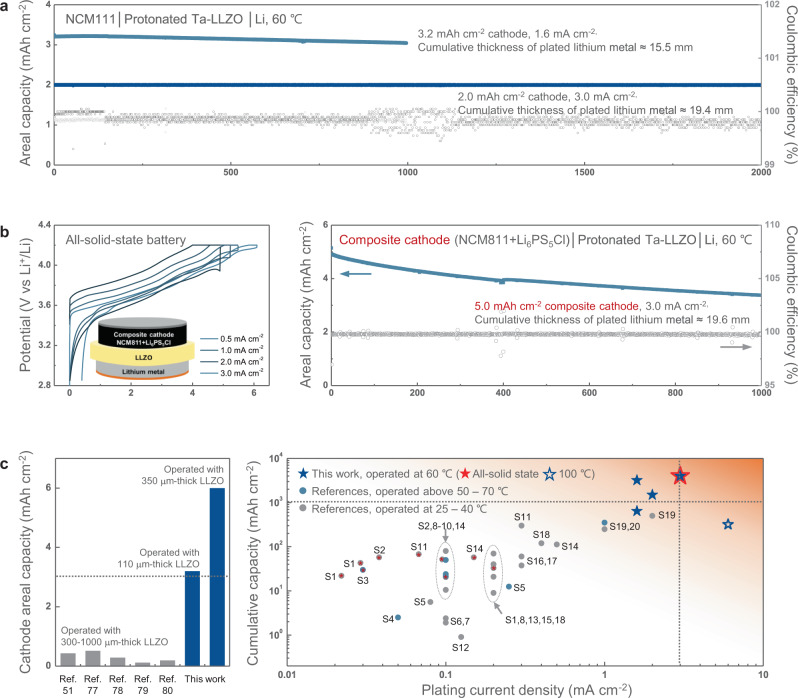
Electrochemical performance of NCM111/protonated Ta–LLZO/Li hybrid cells. **a** Long-term cycling performance. The cells maintained remarkable cycling performance over 1000 and 2000 cycles, delivering 3.2 and 2.0 mAh cm^–2^, respectively, at an average Coulombic efficiency over 99.83%, without significant capacity degradation or short-circuit failure. **b** Voltage profiles and long-term cycling performance of all-solid-state battery using a composite of NCM811 and Li_6_PS_5_Cl electrolyte as the cathode, showing a capacity of 5 mAh cm^–2^ at 60 °C with doped, surface-tailored electrolytes. The cell successfully operated over 1000 times at a high current density of 3 mA cm^–2^ without short-circuiting. **c** Performance comparison of solid‐state batteries using garnet-type solid electrolytes and Li-metal anodes. The left graph shows the cathode capacity of full cells studied previously^51,77–80^ in comparison with the cells of the surface-stabilised Ta–LLZO electrolyte. The right plot summarises the current density and cumulative areal plating capacity at several temperatures based on the previous reports (see Supplementary Table 6 for detailed references, S1–S20 as indicated on the figure) The values corresponding to full cells are distinguished from those of the symmetric cells by red asterisks; the dashed lines indicate the minimum requirement for industrial applications^31^.

To further confirm that interface stabilisation plays an important role in cell stability, we performed long-term cycling tests on the NCM111/protonated Ta–LLZO/Li hybrid cells. Two NCM111 cathodes with capacities of 2 and 3.2 mAh cm^−2^ were used in the cell and cycled at current densities of 3 and 1.6 mA cm^−2^, respectively. Figure 4a suggests that these cells maintained remarkable cycling performance over 2000 and 1000 cycles, respectively, without significant capacity degradation or short-circuit failure for each capacity cell (Supplementary Fig. 30). This corresponds to a cumulative thickness of 19.4 and 15.5 mm, respectively, of plated lithium metal during cell operation. Even after 1000 cycles, the cell had excellent Coulombic efficiency (99.92% at the 1000th cycle). We further examined the long-term performance of the cell using a thinner electrolyte (~110 μm) fabricated via the tape-casting method or by applying a composite cathode with a capacity of 5 mAh cm^−2^ that consisted of Li_6_PS_5_Cl electrolyte and high-nickel NCM811 (LiNi_0.8_Co_0.1_Mn_0.1_O_2_). Supplementary Fig. 31 shows that protonated Ta–LLZO with a thickness of 110 μm was capable of delivering long-term cycle stability over 600 cycles without short-circuiting at a high current density of 2 mA cm^−2^, although it retained slightly less capacity than the thicker pellet due to the different surface properties (Supplementary Fig. 32). In addition, an all-solid-state battery, excluding the ionic liquid electrolyte, was successfully demonstrated using the composite cathode, which could cycle over 1000 times at a high current density of 3 mA cm^−2^ without short-circuiting (Fig. 4b). To the best of our knowledge, this is the first all-solid-state battery that can operate over 1000 cycles, enabled by the garnet-type electrolytes and cathode with a commercially acceptable capacity.

Figure 4c compares the performance of our Li-metal cells with results from the literature for the areal cathode capacity (i.e., equivalent to the amount of Li metal utilised per cycle in a Li-metal cell), cumulative capacity of the deposited lithium, and the operating current density, which are regarded as the important practical parameters for assessing the performance of solid-state Li-metal batteries^31^. The left figure shows that compared with the previously reported studies on garnet-based oxides as a solid-electrolyte, the cell with protonated Ta–LLZO utilised significantly higher cathode capacity (up to 6 mAh cm^−2^) in full cells. The state-of-the-art cells reported to date used cathodes with an areal capacity less than 0.5 mAh cm^−2^, which is hardly applicable to practical battery systems. If we assume our hybrid solid-state battery configuration as a one-unit cell in a pack, the utilisation of the high-capacity-cathode (NCM811) of 6 mAh cm^−2^ would presumably result in a volumetric energy density of 428 Wh L^−1^. Moreover, since we demonstrated the feasibility of the 110-μm-thick Ta–LLZO solid electrolyte with a cathode (NCM111) of 3 mAh cm^−2^ capacity, a new cell would be expected to deliver a remarkable energy density of 470 Wh L^−1^, making it promising for practical applications (see Supplementary Table 5 for the details). The cumulative lithium capacities were calculated and plotted considering the capacity deposited on the Li-metal anode in the full cells (Fig. 4c, right). Except for the symmetric cell reported by Yang^73^ and Taylor^74^, all the previous studies were conducted at current densities below 1 mA cm^−2^, with capacities less than 1 mAh cm^−2^. The cells with the tailored co-doped LLZO electrolyte showed outstanding performance, particularly in terms of the cumulative capacities, which were 4000 mAh cm^−2^ (3200 mAh cm^−2^) when the hybrid cell with a capacity of 2 mAh cm^−2^ (3.2 mAh cm^−2^) was cycled at 3 mA cm^−2^ (1.6 mA cm^−2^). More importantly, the all-solid-state battery cell with the co-doped LLZO electrolyte and 5 mAh cm^−2^ composite cathode (without any liquid) also delivered 4000 mAh cm^−2^ at 3 mA cm^−2^. To the best of our knowledge, these are the highest long-term cycling parameters reported thus far for cells with garnet-oxide electrolytes and Li-metal anodes. This is the first time that a secondary battery of this kind could satisfy the lifespan requirements of energy storage in vehicles and stationary applications^31^. The successful demonstration of the potential applicability of the garnet electrolyte and lithium metal is expected to spur the development of practical all-solid-state batteries with high-energy density and long-lasting cycling stability.

## Methods

### Preparation of the LLZO electrolyte pellets

We prepared Ta-, Al-, Nb-, and Ga,W–LLZO using a solid-state synthesis technique with Li_2_CO_3_ (>99.0%, ChemPoint), La_2_O_3_ (98.6%, MolyCorp), and ZrO_2_ (98%, Zircoa Inc.) as the starting precursors. As dopants, we used Ta_2_O_5_ (99.99%, Sigma Aldrich), Al_2_O_3_ (99.99%, Sigma Aldrich), Nb_2_O_5_ (99.9%, Sigma Aldrich), Ga_2_O_3_ (99.99%, Sigma Aldrich), and WO_2_ (99.9%, Alfa Aesar). La_2_O_3_ was heat treated in N_2_ at 1000 °C for 5 h before use. We mixed the powders at stoichiometry and calcined them in the air at 950 °C for 5 h followed by 1200 °C for 5 h with a heating rate of 150 °C h^−1^. After calcining, the powder was ball milled for 10 min at 300 rpm at the ball-to-powder ratio of 400 wt% by planetary milling (Pulverisette 7, Fritsch, Germany). Ball milling was repeated 12 times with a 5 min interval. The as-prepared powder was mainly a cubic garnet phase along with small amounts of impurity phases, La_2_Zr_2_O_7_ and La_2_O_3_. However, the tetragonal phase was not detected (Supplementary Fig. 2). The particle size distribution was measured by using the particle size analyser (Bluewave, Microtrac). All samples had similar bimodal particle size distributions with peaks at 0.4 and 4 µm, showing approximately the same average particle sizes of 0.7–0.8 µm (Supplementary Fig. 33). We hot-pressed 100 g of calcined powder in a graphite die at 1100 °C for 2 h and 20 MPa in Ar gas with a heating rate of 300 °C min^−1^. The relative density of the obtained pellet was >98% with respect to the theoretical density of LLZO calculated from the XRD data. The pellet was cut using a wire saw into 360-µm-thick slices, which were then laser cut into 14-mm-diameter discs, followed by ultrasonic cleaning in hexane for 10 min and heat treatment at 800 °C for 1 h in an air-controlled box furnace filled with dry air. For the heat treatment, we placed the garnet discs in a Pt container lined with ~100-µm-thick sintered garnet tape of the same composition. The surface of the obtained pellet was polished to a thickness of about 350 µm using polishing machines (LaboForce-3, Struers). We carried out acid treatment for modifying the LLZO surface by simply immersing the discs into a 1 M HCl solution (in distilled water) at a weight ratio of 1:10 (pellet:acid solution) at room temperature. To prevent local variations in the concentration of the acid solution due to the released lithium and/or prevent close contact between the electrolyte and the container, the container was rolled at about 60 rpm during protonation. We then removed the solution, washed the discs with ethanol, and dried them in a dry room.

### Colouration tests

To prepare a flat, smooth surface, we carefully polished the surface of a prepared pellet with P800-, 1200-, 2400-, and 4000-grit SiC abrasive paper to a thickness of ~300 μm. The last two steps were performed in an Ar glove box to minimise surface contamination from exposure to air. Then, we attached the lithium metal, scratched using a brush to expose the fresh lithium metal surface, to the prepared LLZO, and applied pressure of 250 MPa to the assemblies using a cold-isostatic press to effectively adhere the lithium metal foil to the LLZO. After assembly, we placed each pellet on a part of a 2032-coin cell and heated them up to 200 °C over a period of 10 min on a hot plate. (Molten metal is commonly applied directly to a ceramic pellet for chemical colouration tests. However, this can crack the pellet owing to thermal shock, and the contact of the molten lithium with LLZO can vary depending on the surface condition of LLZO^19^.).

### Characterisations of the solid electrolyte

We characterised the crystal structures of the LLZO electrolytes using XRD. Diffraction patterns of the LLZO pellets before and after the colouration test (Fig. 1b and Supplementary Fig. 15), the as-prepared powder (Supplementary Fig. 1 for as-prepared powder and 2) and the tape-cast LLZO electrolyte (Supplementary Fig. 32) were collected using a PANalytical (Empyrean) diffractometer with Cu Kα radiation (λ = 1.5406 Å). The data were recorded in the 2θ range of 10°–90°, with a step size of 0.02° and a step time of 4.5 s. XRD patterns for the original pristine pellets, protonated pellets and the crushed pellets (Fig. 2d and Supplementary Fig. 1 for pristine pellet and 18–21), which were subjected to Rietveld refinement, were collected on another PANalytical (Empyrean) diffractometer equipped with a monochromator and Cu Kα radiation (λ = 1.5406 Å) to obtain the high resolution to precisely deconvolute the overlapped patterns arising from the coexistence of the two LLZO phases (cubic vs. tetragonal phases). The data were recorded in the 2θ range of 10°–120°, with a step size of 0.01313° and a step time of 1.6 s. In order to prevent the exposure of the electrolytes to the ambient atmosphere, all pellet samples were examined in an air-tight holder. We conducted XPS with a Quantum 2000 Scanning ESCA Microprobe (Physical Electronics, Inc.) spectrometer using focused monochromatised Al Kα radiation (1486.6 eV). The residual pressure inside the XPS analysis chamber was 9.3 × 10^−10^ Pa. To avoid any contamination, we transferred the LLZO pellets from the Ar-filled glove box to the XPS chamber using a specially designed air-proof chamber. We examined the surface morphology and cross-sectional microstructure of the acid-treated LLZO electrolytes using an SU-8030 FE-SEM (Hitachi) coupled with an EDS spectrometer with a 5 kV accelerating voltage and an 8 mm working distance. We evaluated the mechanical strength according to the ring-on-ring test using an MTS 10D Load Frame, with a 1.9 cm diameter support ring and 0.635 cm diameter load ring, both available from Sintech Corporation. We prepared cross-sectional samples for TEM at the LLZO surfaces using a focused ion beam (FIB, FEI-Helios 450-F1) and finally milled them with Ga ions at a 5 kV acceleration voltage. We acquired bright-field TEM images and SAD patterns to identify the phase and crystallinity of LLZO near the surface using double Cs-corrected TEM (FEI Titan cubed 60-300). We conducted TEM with minimum e-beam exposure conditions because LLZO is prone to transform into its amorphous phase under strong e-beam irradiation.

### XRD refinement

Phase fraction of each compound was obtained by Rietveld refinement using the crystallographic information file. The Thompson-Cox-Hastings pseudo-Voigt function was applied to determine the shape of the diffraction peak. Rietveld refinement was conducted for the phase fraction analysis of the XRD patterns of the pellet samples using the Fullprof program^75,76^. The detailed sample characterisations can be found in Supplementary Figs. 18–21.

### Electrochemical characterisation

We prepared symmetric cells with 14-mm diameter pellets that were polished to remove the surface contamination to the maximum extent possible, using P800-, 1200-, and 2000-grit SiC abrasive paper, or protonated as demonstrated in the previous section in a dry room, where the dew point was maintained under −50 °C. In either case, prepared pellets were rinsed with ethanol and dried in a dry room, and any remaining dust was blown using a high-pressure N_2_ gas gun. Lithium metal electrodes, 100-µm-thick lithium metal on 10-µm-thick Cu foil (Honjo Metal Co., Ltd.) with a diameter of 8 or 11 mm, were placed onto both sides of the pellet, and the assembly was vacuum sealed inside a polymer-coated aluminium pouch, which is commonly used for battery fabrication. Then, Li-metal anodes were attached by applying a pressure of 250 MPa for 3 min using a cold-isostatic press to improve the physical contact between the LLZO pellet and Li-metal anode. We also prepared asymmetric cells with a blocking gold electrode sputtered onto one side of the LLZO pellet and a non-blocking Li-metal electrode on the opposite side, which was attached in the same manner as the symmetric cell electrodes. Finally, we assembled the cells applying our modified 2032-type coin cell configuration. The cells were constructed as a conventionally structured coin cell, except for the spring used to apply the pressure. Instead, we vacuum sealed the cells in an aluminium pouch, with extended lines for electrical connection. With this configuration, we could prevent air exposure and apply a constant atmospheric pressure by maintaining the vacuum state. Using these cells, we performed the EIS measurements, galvanostatic cycling tests, and critical current density measurements. EIS measurements were conducted at 60 and 25 °C at an open-circuit voltage in the galvanostatic mode over a frequency range of 0.1–10 kHz using an alternating current perturbation of 10 mV, using a frequency response analyser (Solartron, SI 1255 FRA) in conjunction with a potentiostat (Solartron, SI 1287 ECI). Galvanostatic cycling tests were conducted on the symmetric cells at 60 and 25 °C at a current density of 0.2 mA cm^−2^ with 1 h of Li plating/stripping. The critical current density measurements were performed for pristine and protonated Ta–LLZO and Al–LLZO solid electrolytes at 60 and 25 °C. The cells employed 100-µm-thick Li metal on 10-µm-thick Cu foil with a diameter of 11 mm (0.95 cm^2^) for the tests at 60 °C or 8 mm (0.50 cm^2^) for the tests at 25 °C, and they were cycled twice with 30 min of lithium plating/stripping at each current density. The current density was increased from 0.1 to 1 mA cm^−2^ at a step size of 0.1 mA cm^−2^ and from 1.0 to 3.0 mA cm^−2^ at a step size of 0.2 mA cm^−2^. To evaluate the electrochemical performance of the LLZO solid electrolyte in a battery full cell using a Li-metal anode, we introduced a hybrid electrolyte cell in which an ionic liquid electrolyte was used as the cathode electrolyte (catholyte) and a solid oxide electrolyte was used as the Li-metal anode electrolyte (anolyte), as shown in Fig. 3a. For the dual-structured electrolytes, a full cell with a high-loading cathode (active material ≥93 wt%) was fabricated via infiltration with the liquid catholyte, and microscale short circuits were clearly detected by monitoring the potential transient curves under galvanostatic charging mode. We used coin cells (20 mm diameter) in all full-cell measurements. We fabricated the hybrid electrolyte cells in a dry room using an ionic liquid as the catholyte and an LLZO solid electrolyte as the anolyte. First, 20-µm-thick Li metal on 10-µm-thick Cu foil (Honjo Metal Co., Ltd.) was attached to the protonated surface of the LLZO pellet by cold-isostatic pressing at 250 MPa. We used a commercially available NCM111 electrode (loading capacity: 3.2 g cm^−3^, active material: 93 wt%; Samsung SDI) coated onto Al foil as the cathode. The ionic liquid Pyr13FSI (Kanto Chemical Co. Inc.) was mixed with LiFSI salt (2 M) to prepare the catholyte. We dropped the mixed solution onto the cathode and then infiltrated it into the cathode under a vacuum for 2 h. The infiltrated amount of ionic liquid was 20 wt% relative to the cathode weight. We placed the infiltrated cathode on the other side of the LLZO pellet in a 2032-coin cell. To eliminate the possibility of direct contact between the ionic liquid and Li-metal, we used a relatively small cathode (0.4 cm in diameter) for the hybrid electrolyte cell. Finally, we sealed the cell under a vacuum using a pouch cell. The charge/discharge characteristics of the hybrid electrolyte cells were examined at 60 and 100 °C using a battery cycler (TOSCAT-3100, Toyo System). The cells were charged using a conventional constant current (CC)–constant voltage (CV) protocol and discharged in CC mode in the potential range of 2.85–4.2 V (vs. Li^+^/Li). The electrochemical profiles at 60 °C were obtained by increasing the current density stepwise; i.e., the cells were charged/discharged with current densities of 0.3 mA cm^−2^ for the first cycle, 0.5 mA cm^−2^ for the next five cycles, 1 mA cm^−2^ for the subsequent five cycles, 1.6 mA cm^−2^ for the 12th to the 21st cycle, 2 mA cm^−2^ for the 22nd to the 31st cycle, and 3 mA cm^−2^ for the 32nd to the 41st cycle. For the rate capability test at 100 °C, the cells were operated with increasing current densities. The cells were cycled at 0.3 mA cm^−2^ for the first cycle and then the current densities are increased from 0.5 and 1 to 1.6 mA cm^−2^ and then increased from 2.0 to 10.0 mA cm^−2^ at a step size of 1.0 mA cm^−2^. The cells were cycled five times at each current density. For data reliability, we evaluated five cells for each electrolyte. To further confirm the interface stabilisation effect on the cell performance, we conducted long-term cycling tests on the NCM111/protonated Ta–LLZO/Li hybrid cells using two NCM111 cathodes with capacities of 2 and 3.2 mAh cm^−2^. With 2 mAh cm^−2^ cathode, the cells were charged/discharged with current densities of 0.3 mA cm^−2^ for the first cycle, 0.5 mA cm^−2^ for the next two cycles, 1 mA cm^−2^ for the subsequent two cycles, and cycled at current densities 3 mA cm^−2^ for 2000 times. With 3.2 mAh cm^−2^ cathode, the cells were charged/discharged with current densities of 0.3 mA cm^−2^ for the first cycle, 0.5 mA cm^−2^ for the next five cycles, 1 mA cm^−2^ for the subsequent five cycles, and cycled at current densities 3 mA cm^−2^ for 1000 times.

## Supplementary information

Supplementary information

## Data Availability

The data generated or analysed in this study are provided in the Supplementary information/Source Data file (CIF files used for DFT calculation) and available from the corresponding author on reasonable request.
