# Supplementary material for: High-energy and durable lithium metal batteries using garnet-type solid electrolytes with tailored lithium-metal compatibility
Source: Nat Commun. 2022 Apr 6;13:1883. doi: 10.1038/s41467-022-29531-x (PMC8986853; doi:10.1038/s41467-022-29531-x)
Supplement: Supplementary file 1 — Supplementary information [file 41467_2022_29531_MOESM1_ESM.pdf]

Supplementary Information

High-energy and durable lithium metal batteries using garnet-type solid electrolytes with tailored lithium metal compatibility

Sewon Kim[1,2†], Ju-Sik Kim[2†], Lincoln Miara[3], Yan Wang[3], Sung-Kyun Jung[2], Seong Yong Park[4], Zhen Song[5], Hyungsub Kim[6], Michael Badding[5], JaeMyung Chang[5], Victor Roev[2], Gabin Yoon[2], Ryounghee Kim[2], Jung-Hwa Kim[4], Kyungho Yoon[1], Dongmin Im[2*], Kisuk Kang [1,7,8,9,10*]*

Supplementary Note 1: Electrochemical impedance spectroscopy (EIS) experiment

We performed EIS experiments with asymmetric cells. A blocking gold electrode was sputtered onto one side of the LLZO pellet and a non-blocking lithium metal electrode was sputtered onto the other side using a cold isostatic press under a pressure of 250 MPa. We recorded the spectra at 25 °C and 60 °C at an open-circuit voltage in the galvanostatic mode over a frequency range of 0.1 Hz – 10 kHz. An alternating current (AC) perturbation of 10 mV was applied and a frequency response analyser (Solartron, SI 1255 FRA) in conjunction with a potentiostat (Solartron, SI 1287 ECI) was used to conduct the experiments. We recorded the spectra at various time intervals and compared the spectra recorded for each LLZO pellet to understand how the reactivity of LLZO with lithium varies with composition. The intercept on the real impedance axis in the high-frequency region corresponds to the resistance of the bulk LLZO electrolyte. The low-frequency semi-circle with a tail results from the interfacial resistance at the LLZO/Li electrode interface and the capacitance at the LLZO/Au electrode interface.[1] Note that the interfacial impedance estimated from the EIS spectra solely represents the chemical reactions at the interface over time because we applied a small potential perturbation (10 mV) at the open-circuit voltage. This implies that significant lithium plating (or stripping), which affects the physical contact conditions, did not occur when EIS experiments were conducted.

Supplementary Note 2: Density functional theory (DFT) calculations

The structures of the doped garnets (Ta, Al, Nb, Ga, W, and H) were determined from the original experimentally determined structure of $Li_7La_3Zr_2O_{12}$. Metal dopants were introduced at appropriate sites (i.e., Ta, Nb, and W in Zr sites; Al and Ga in Li sites). The lithium ions were removed as vacancies from the highest energy sites to maintain charge neutrality. In the protonated structures, lithium ions were partially substituted by hydrogen ions. The electrostatic energy criterion was considered for the analysis performed using the Python Materials Genomics (pymatgen) package[2] to pre-screen structures with Li^{+}/Al^{3+}(Ga^{3+})/vacancy and Ta^{5+}(Nb^{5+}, W^{5+})/Zr^{4+} orderings to obtain the lowest-energy structure. We then performed structural optimization experiments and total energy calculations using the DFT method with the Perdew–Burke–Ernzerhof (PBE) generalised-gradient approximation (GGA),[3] implemented in the Vienna Ab initio Simulation Package (VASP).[4] We used the projector augmented wave potentials with a kinetic energy cut-off of 520 eV and a k-point grid of $\geq 500/n_{atoms}$ for all the calculations.

We evaluated the electrochemical stability from grand canonical phase diagrams following reported protocols.[5,6] The oxidation and reduction limits of the stability window were defined as the voltage limits at which the material begins to get oxidised via the process of lithium extraction or reduced via the method of lithium insertion, respectively. To determine the relevant phase diagrams, we obtained the DFT total energies of all the related compounds in the given quintenary system of Li−La−Zr−O−M (where M = dopant) from the Materials Project database [www.materialsproject.org]. The data presented in the database were computed using the same DFT method that we had followed.

Supplementary Note 3: Other possible origins of the strengthening

Although we suggested the strain release that accompanies acid treatment, which effectively removes the secondary garnet phase in the pellet as a discernible origin of the enhancement of the mechanical strength, there can be additional explanations about the strengthening mechanism regarding on the protonation, the substitution of lithium ions in LLZO to protons. It is speculated that a compressive stress introduction on the electrolyte surface by the protonation could increase the strength. It has been reported that the replacement of small alkali ions in the original glasses by guest ions, without changing the network structure, induces a compressive stress on the surface, which increases the mechanical strength of the glass materials.[7,8] This infers that the mechanical strength of the LLZO pellets may have been enhanced in the similar mechanism. That is, since the lattice parameters of LLZO can increase through the exchange of lithium ions in the LLZO with protons, as reported in the previous research by Liu et al,[9] it can cause the compression stress in the surface region of the garnet pellet as in the glass strengthening. As a result, the mechanical strength could have been improved. Although the lattice constant increases have been hardly observed in our bulk XRD analysis of the pellet LLZO, it is difficult to exclude the possibility that mechanical strength strengthening through this mechanism has occurred, and further investigation is required in the future. Besides, protonation can affect the strain release as well by inducing the lithium disordering that leads the phase transition from tetragonal phase to cubic phase.[10-12]

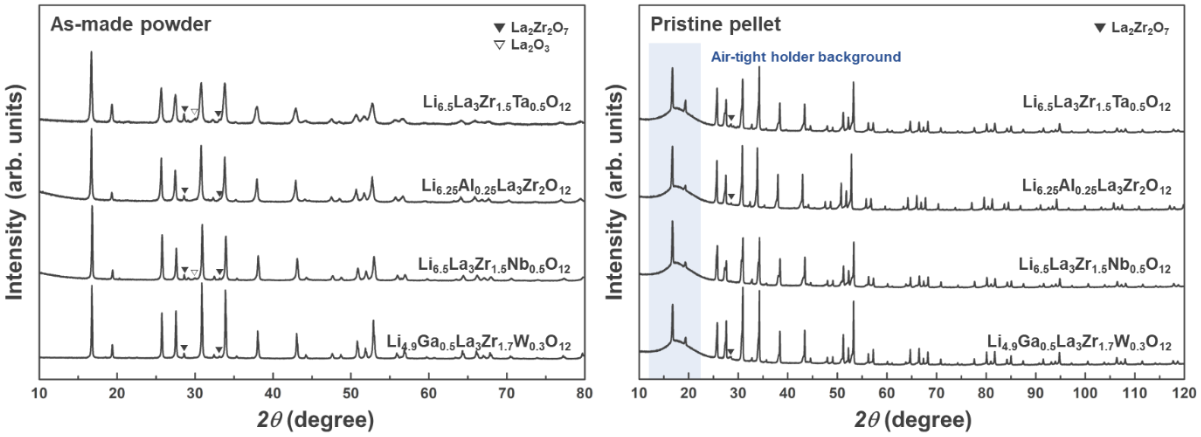

Supplementary Figure 1. X-ray diffraction (XRD) patterns of as-prepared powders and the pellets of doped-LLZO with various dopants. All the powder samples retain the cubic LLZO phase as a main phase whereas all pellets consist of cubic and tetragonal LLZO phases. Regardless of the form of LLZOs, trace amounts of impurity phases, such as $La_2Zr_2O_7$ and La_2O_3 are detected. (Rietveld refinement results are provided in Supplementary Figure 2 and 18).

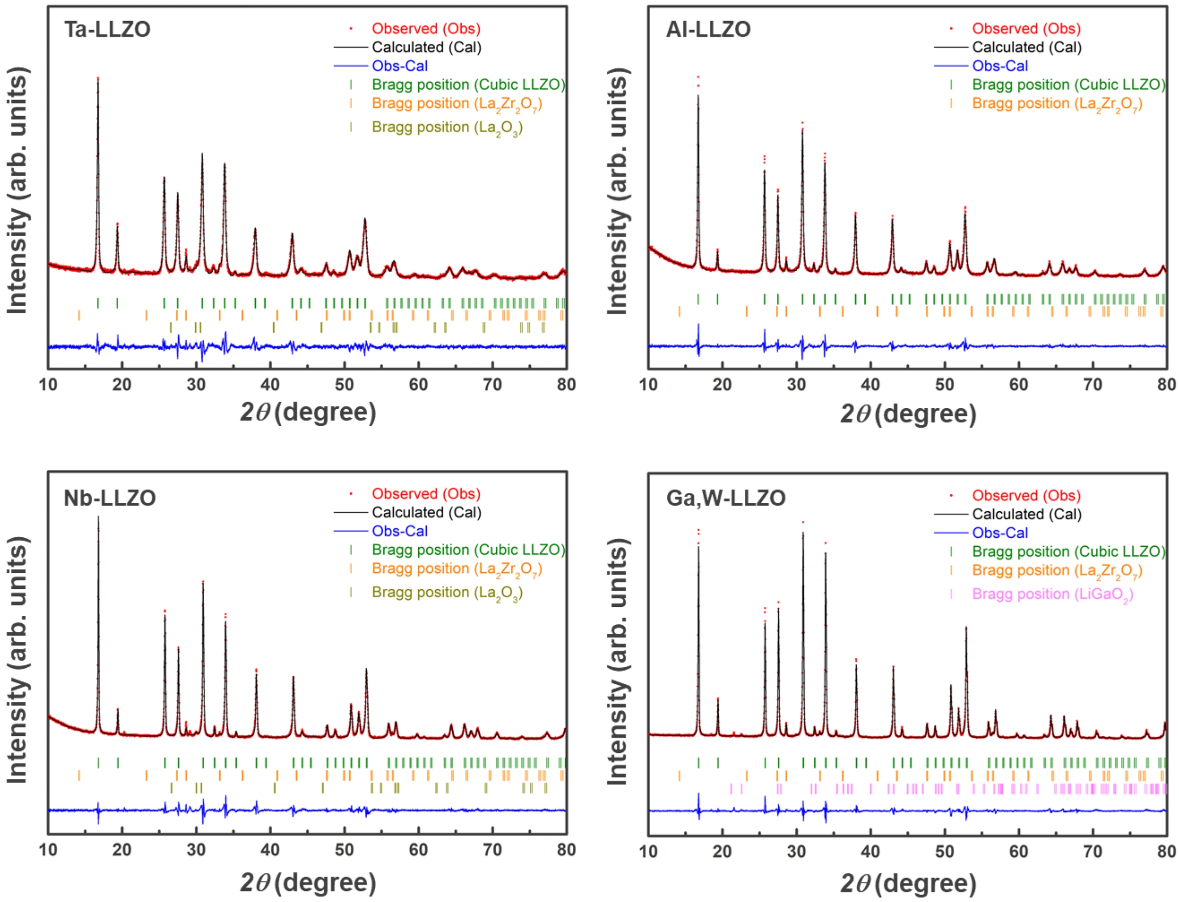

Sample	Phase	Lattice parameter (a, Å)	Cell volume ($Å^3$)	Phase fraction (wt.%)	R_I(%)	R_F(%)
$Li_{6.5}La_3Zr_{1.5}Ta_{0.5}O_{12}$ (R_p: 5.21 %, R_{wp}: 6.58 %)	Cubic	12.9816 (4)	2187.7 (1)	96.3 (7)	2.30	1.83
	$La_2Zr_2O_7$	10.80732	1262.275	2.88 (7)		
	La_2O_3	3.93367 (6.13873)	82.263	0.82 (3)		
$Li_{6.25}Al_{0.25}La_3Zr_2O_{12}$ (R_p: 3.37 %, R_{wp}: 4.47 %)	Cubic	12.9894 (2)	2191.63 (5)	97.6 (6)	1.63	1.64
	$La_2Zr_2O_7$	10.80732	1262.275	2.42 (6)		
	La_2O_3					
$Li_{6.5}La_3Zr_{1.5}Nb_{0.5}O_{12}$ (R_p: 4.38 %, R_{wp}: 6.11%)	Cubic	12.9344 (2)	2163.92 (6)	97.7 (6)	2.32	1.76
	$La_2Zr_2O_7$	10.80732	1262.275	1.98(7)		
	La_2O_3	3.93367 (6.13873)	82.263	0.33(4)		
$Li_{4.9}Ga_{0.5}La_3Zr_{1.7}W_{0.3}O_{12}$ (R_p: 5.09 %, R_{wp}: 6.59 %)	Cubic	12.9447 (1)	2169.10 (4)	97.0 (5)	2.20	3.00
	$La_2Zr_2O_7$	10.80732	1262.275	2.49 (6)		
	$LiGaO_2$	3.93367 (6.13873)	82.263	0.5 (1)		

* For the reliability of the data, only scale factor was set as a refinement parameter for the impurity phases.

Supplementary Figure 2. XRD patterns and Rietveld refinement results of the as-prepared LLZO powders doped with various metals such as Ta, Al, Nb, and Ga,W. Each powder sample shows a cubic garnet phase as a main phase with a small amount of impure phases, such as $La_2Zr_2O_7$ (about 2~3 % for all compositions), La_2O_3 (less than 1% for Ta-LLZO and Nb-LLZO) and $LiGaO_2$ (about 0.5% for Ga,W-LLZO).

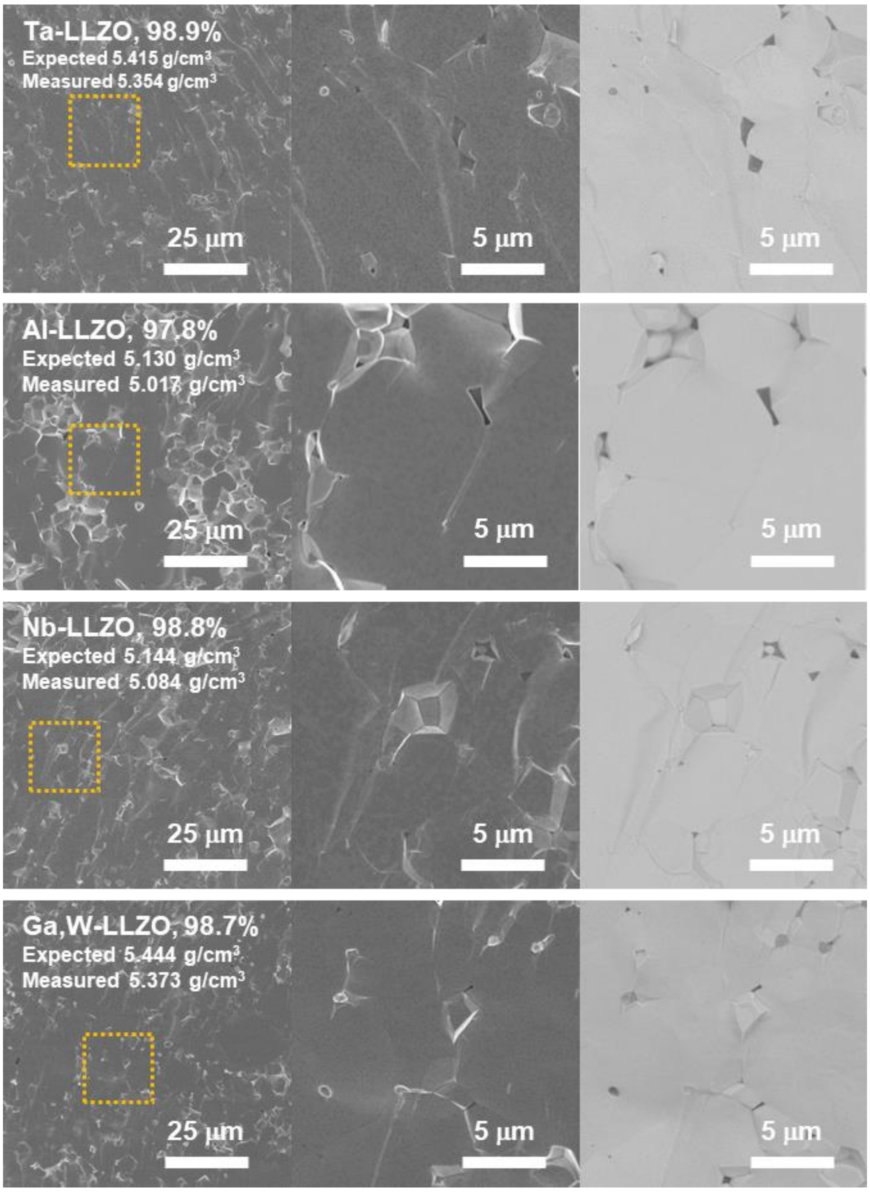

Supplementary Figure 3. Cross-sectional scanning electron microscopy (SEM) images of the fractured LLZO pellets with various dopants (Ta, Al, Nb, Ga,W). The SEM technique was used to analyse the fractured surface of each pellet, and the microstructures were characterised. All samples exhibited dense microstructures consistent with the relative densities (shown in the images) calculated from the measured densities using Archimedes' principle in anhydrous isopropanol and the expected densities estimated based on the XRD refinement results. Al-doped LLZO exhibited a partial intergranular fracture with an average grain size of 5 µm. The other doped LLZOs exhibited similar microstructural features. The average grain

sizes (approximately 3 μm) were comparable. A major fracture mode (primarily the transgranular mode) was observed, indicating a high grain boundary strength.

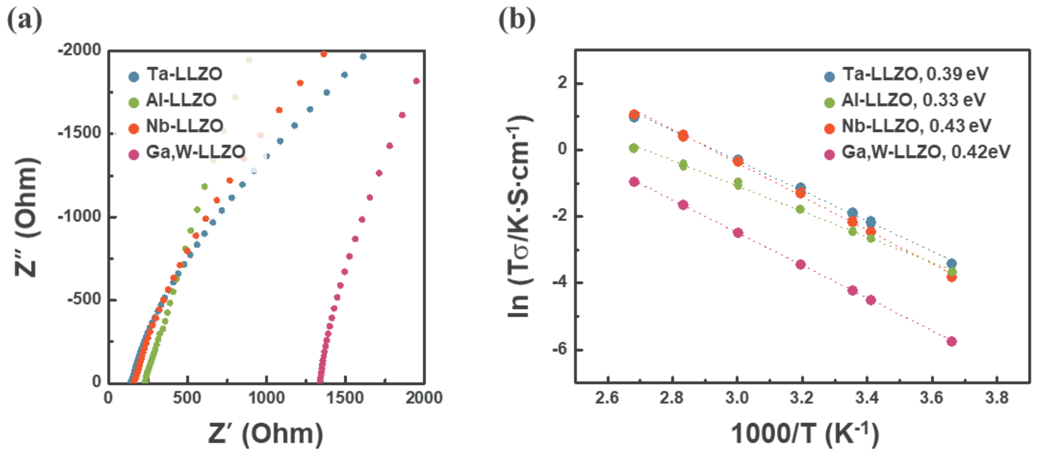

Supplementary Figure 4. (a) EIS profiles of Au/LLZO/Au symmetric cells at 25 °C and (b) Arrhenius plot of the total ionic conductivities of the LLZO pellets doped with various metals (Ta, Al, Nb, and Ga,W). AC-impedance measurements using Au (blocking) electrodes were performed on polished pellets (~14 mm in diameter and 300–350 μm in thickness) to determine the total ionic conductivities. As lithium metal can react with Nb- or Ga,W-LLZO during conductivity measurements and affect the total resistance, we used the Au electrode to conduct the experiments with the LLZO samples. The electrode area was 0.95 cm^2. We carried out the experiments in the frequency range of 1 MHz – 0.1 Hz at an amplitude of 10 mV (due to equipment limitations). We observed only one partial semi-circle at the high-frequency and a diffusion spike at the low-frequency regions of the Nyquist plots. As we could not resolve the bulk and grain-boundary conductivities (as reported in previous studies)[13,14], we calculated the ionic conductivities using the intercept values of the real axis of the Nyquist plots. The calculated ionic conductivities of Ta-, Al-, Nb- and Ga,W-LLZO at 25 °C were 0.51, 0.29, 0.39, and 0.05 mS cm^{-1}, respectively. The corresponding activation energies are presented in the figure. These values agree well with the reported values.[15]

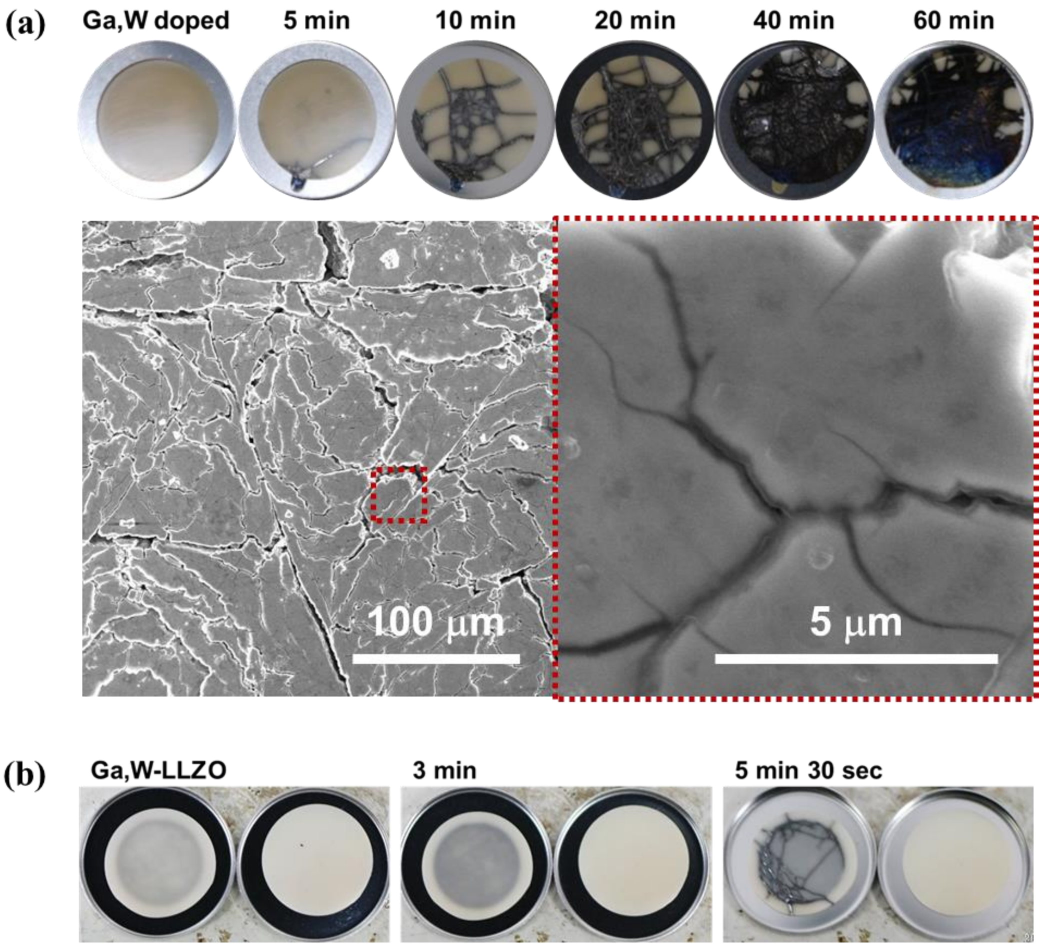

Supplementary Figure 5. (a) Optical images of Ga,W-LLZO pellets in contact with lithium metal (recorded over time at 200 °C) and the corresponding SEM images of the surface exhibiting numerous cracks and the pulverization of the pellet and (b) Comparison between the images of a Ga,W-LLZO pellet in contact with lithium metal and the images of another pellet where the lithium metal contact at 200 °C was absent. The results indicated that the origin of pulverization could be attributed to the chemical reaction occurring between LLZO and lithium metal, not simply to the thermal shock. The volume change and the corresponding strain induced by the chemical reactions between the electrolyte and lithium metal can cause the fracture of these pellets, as reported by Tippens *et al.*.[16] It was reported that the growth of an interphase (the $Li_{1+x}Al_xGe_{2-x}(PO_4)_3$ (LAGP) and lithium metal interface) during the electrochemical cycling process results in the formation of fracture in the

material.

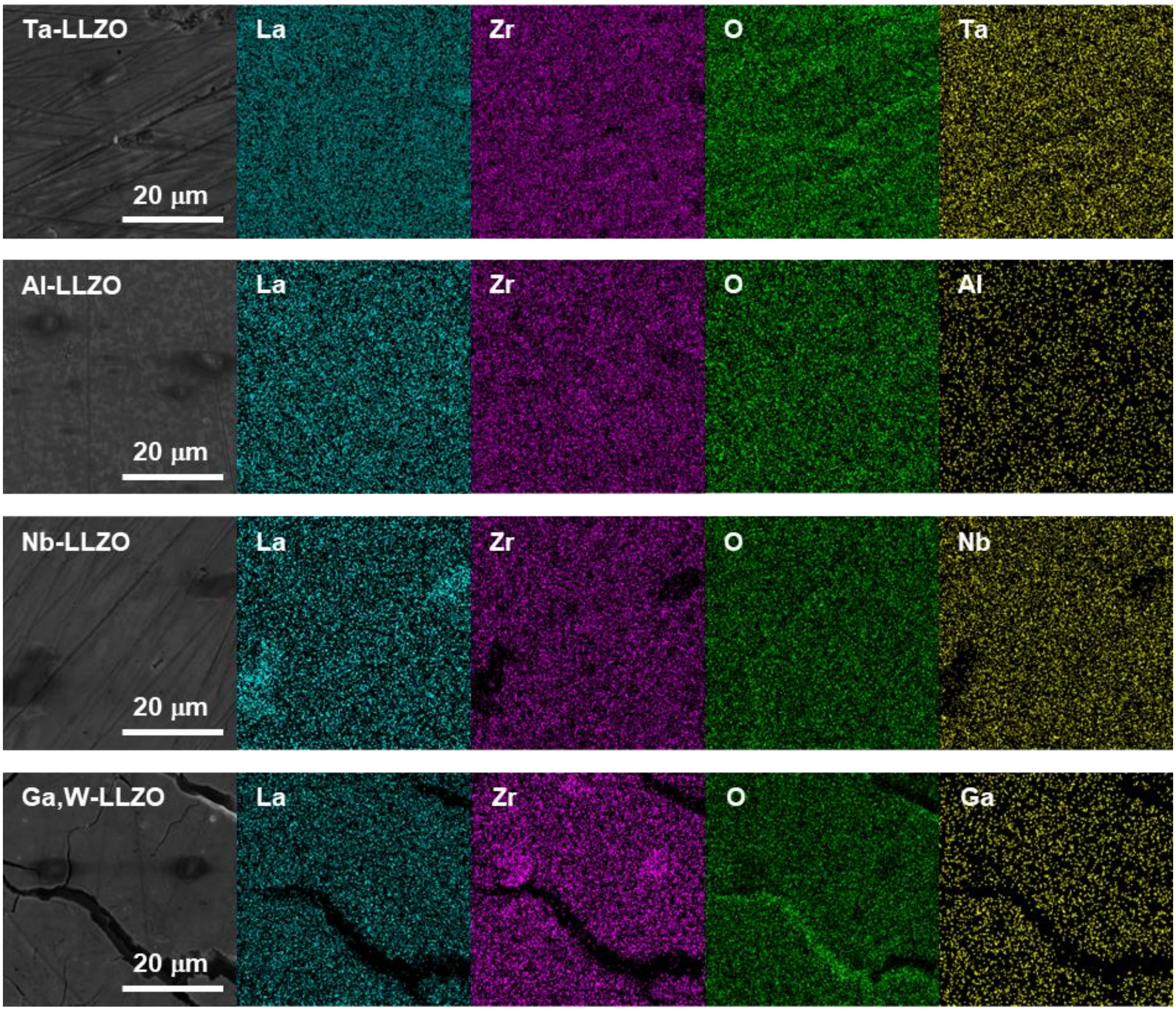

Supplementary Figure 6. SEM images and energy-dispersive X-ray spectroscopy (EDS) images recorded for the LLZO surfaces that were in contact with the lithium metal during the colouration test. Cation segregation on the surface of Nb- and Ga,W-LLZO pellets were observed. Transition metals did not noticeably segregate from the Ta- and Al-LLZO surfaces, whereas a non-uniform distribution of La or Zr was observed in the case of Nb- and Ga,W-LLZO, indicating that it rapidly reacted with lithium metal during the colouration test.

(a)

(b)

(c)

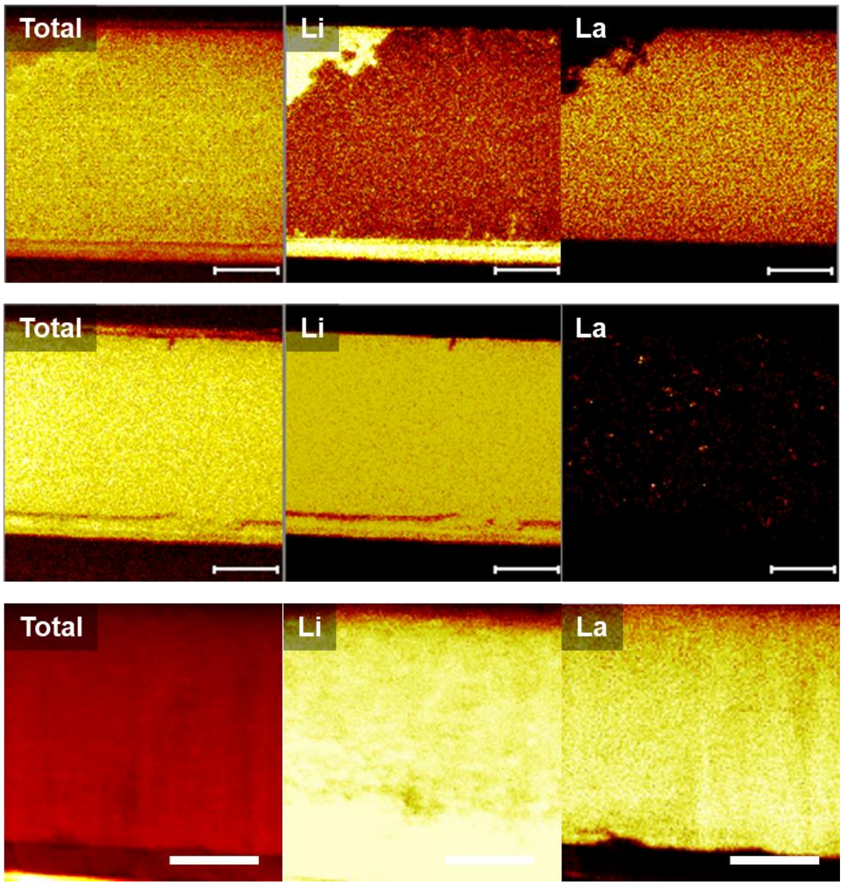

Supplementary Figure 7. Comparison of the cross-sectional SIMS images of (a) Ta-LLZO, (b) Nb-LLZO, and (c) Ga,W-LLZO. The images were recorded after the colouration test had been conducted (Scale bars: 100 μm). The amount of lithium present in Ta-LLZO was significantly less than that present in lithium metal (100% lithium content). The lithium contents in Nb-LLZO and Ga,W-LLZO were as high as the lithium content in the lithium metal over the entire area, making it difficult to distinguish the electrolyte from lithium metal. This implies a favourable reaction between Nb-LLZO (and Ga,W-LLZO) and the lithium metal.

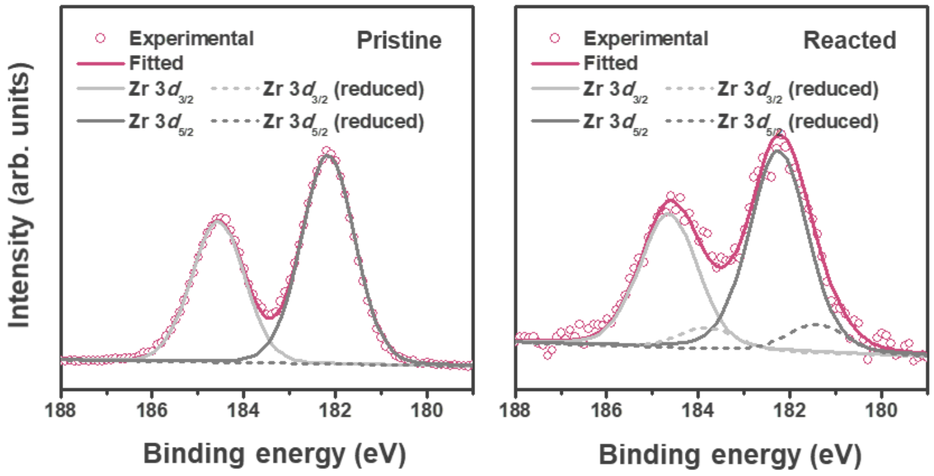

Supplementary Figure 8. X-ray photoelectron spectroscopy (XPS) profiles recorded for the cross-section of Ga,W-doped LLZO pellet before and after colouration tests. Zr 3*d* core-level spectra before (left) and after (right) the reaction. Fitted curves obtained using fixed spin splits ($3d_{3/2}$ - $3d_{5/2}$ = 2.40 eV) are presented. Two different chemical environments around Zr were observed in the pristine pellet. A doublet signal arising from those two peaks (separated by 2.40 eV) was observed in the profile of the reacted pellet, indicating the reduction of Zr.

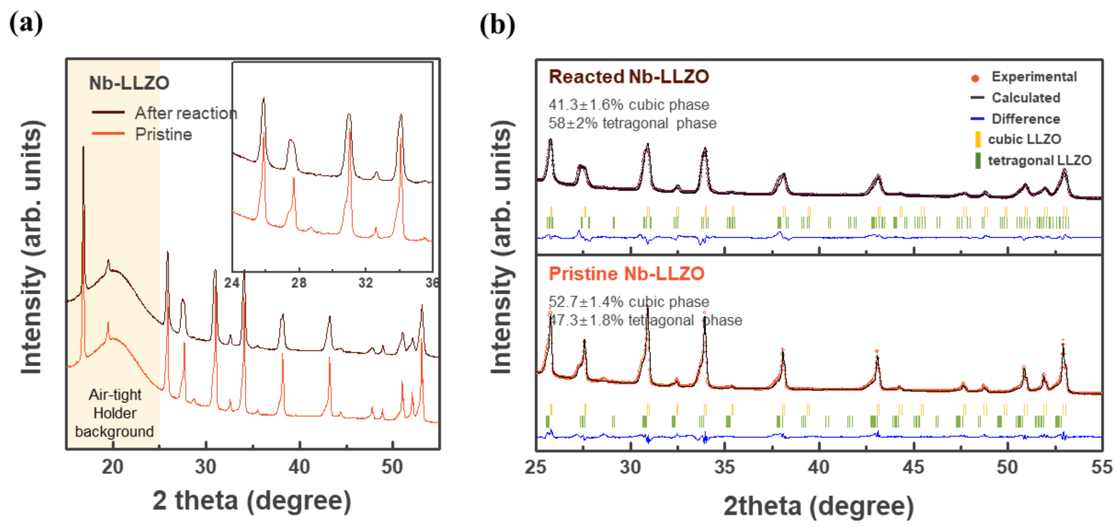

Sample	Phase	Lattice Parameters (*a*, Å)	Cell vol.[Å^3]	Phase Fraction (wt.%)	c/a	R_I(%)	R_F(%)
Pristine Nb-LLZO R_p: 2.59 % R_{wp}: 4.22 %	Cubic	12.93623 (11)	2164.83 (3)	52.7 (14)	1	4.40	3.14
	Tetra	13.0380 (12)	2204.8 (5)	47.3 (18)	0.995	7.67	3.82
Reacted Nb-LLZO R_p: 3.02 % R_{wp}: 5.22 %	Cubic	12.9277 (3)	2160.6(1)	41.3 (16)	1	4.30	3.18
	Tetra	13.0261 (6)	2179.4 (2)	58 (2)	0.986	11.2	6.07

Supplementary Figure 9. XRD patterns and Rietveld refinement results obtained for the Nb-LLZO pellets. The patterns and data were recorded before and after the colouration test. It was observed that the relative phase amounts (cubic and tetragonal phases) and the lattice parameters (after the colouration tests) changed after the reaction. The relative amount of tetragonal phase increased and the c/a ratio decreased after the reaction, indicating that Nb-LLZO exhibited more tetragonal-like phase. We believe that this result provides insight into the reaction between lithium metal and Nb-LLZO. Lithium is introduced into the Nb-LLZO system via an Nb^{5+} reduction process. Thompson, T. *et al.*[17] reported that Ta-doped LLZO exhibited the tetragonal phase under conditions of low lithium vacancy. We hypothesised that lithium insertion, accompanied by Nb^{5+} reduction, can potentially induce the structural change of Nb-LLZO, which appeared as a change in the XRD pattern (after the reaction with lithium metal).

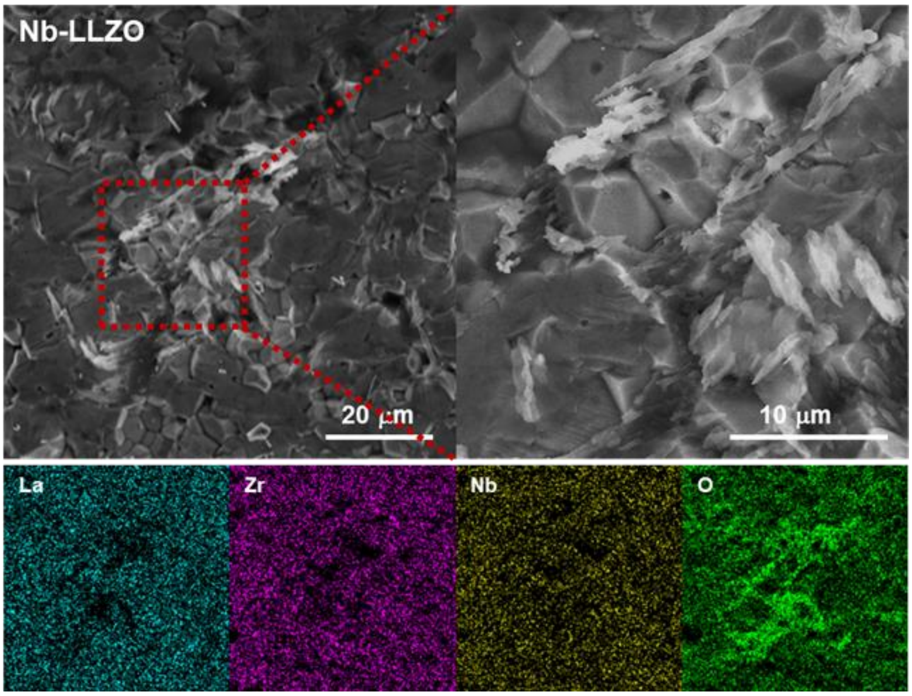

Supplementary Figure 10. EDS images of the cross-section of Nb-LLZO pellet recorded after the colouration test. Lithium metal propagation through the grain boundaries is observed.

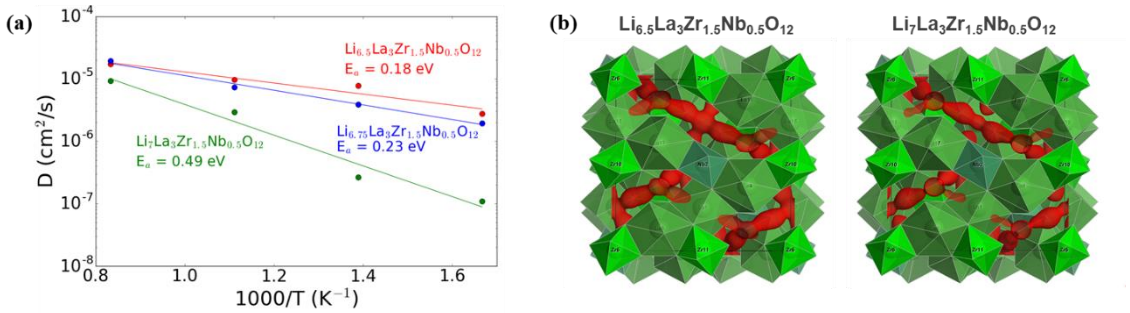

Supplementary Figure 11. (a) Calculated Arrhenius relations of lithium ion diffusivities for $Li_{6.5}La_3Zr_{1.5}Nb_{0.5}O_{12}$, $Li_{6.75}La_3Zr_{1.5}Nb_{0.5}O_{12}$, and $Li_7La_3Zr_{1.5}Nb_{0.5}O_{12}$ and **(b) Calculated lithium ionic probability densities in $Li_{6.5}La_3Zr_{1.5}Nb_{0.5}O_{12}$ and $Li_7La_3Zr_{1.5}Nb_{0.5}O_{12}$.** The ionic conductivities in Li-excess garnets doped with Nb^{4+} ($Li_{6.75}La_3Zr_{1.5}Nb_{0.5}O_{12}$ and $Li_7La_3Zr_{1.5}Nb_{0.5}O_{12}$) are much lower than the ionic conductivities in $Li_{6.5}La_3Zr_{1.5}Nb_{0.5}O_{12}$ doped with Nb^{5+}. The Li^+ migration channels are well connected in $Li_{6.5}La_3Zr_{1.5}Nb_{0.5}O_{12}$, but the channels are disrupted in $Li_7La_3Zr_{1.5}Nb_{0.5}O_{12}$ near the Nb^{4+} sites. It was speculated that the bulk resistance of Nb-doped LLZO increases with time in Figure 1c because the lithium vacancy concentration decreases with an increase in Li^+ in the Nb-doped LLZO due to the reduction of Nb^{5+}. It is supported by the *ab initio* Molecular Dynamics (AIMD) results here, which show that the ionic conductivities of $Li_{6.75}$- or Li_7- garnet compositions with Nb^{4+} are significantly lower than that of $Li_{6.5}La_3Zr_{1.5}Nb_{0.5}O_{12}$ with only Nb^{5+} due to the disrupted Li^+ migration channels near Nb^{4+}.

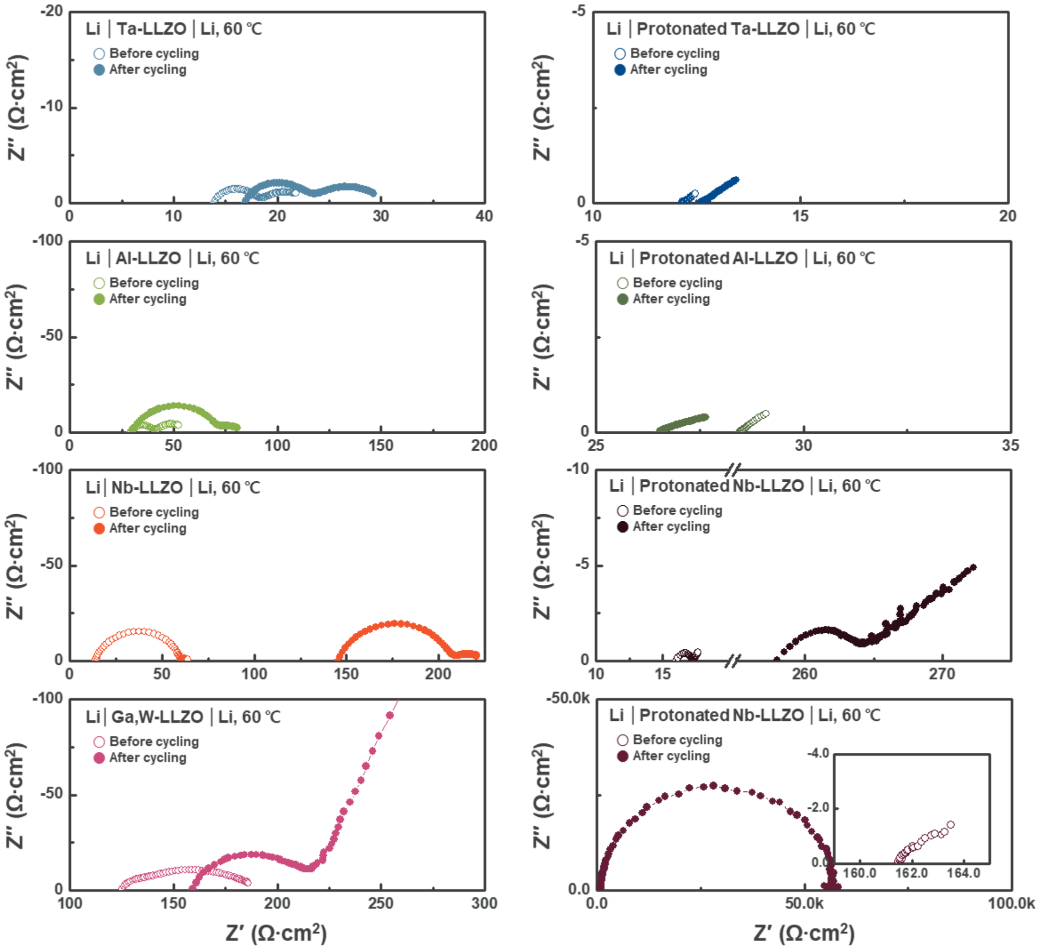

Supplementary Figure 12. EIS profiles and the results obtained from experiments conducted (before and after galvanostatic cycling) with Li/LLZO/Li symmetric cells at 60 °C (Figure 1d and Supplementary Figure 15d). Analysis of the EIS profiles revealed slight changes in the bulk or interfacial resistance (before and after cycling) in the cells fabricated using Ta- or Al-LLZO (that exhibited stable cycling performance). The results agreed well with the results obtained from the galvanostatic cycling experiments. Significant changes in both bulk and interfacial resistance were observed in cells fabricated using Nb- or Ga,W-LLZO (that exhibited unstable cycling behaviour).

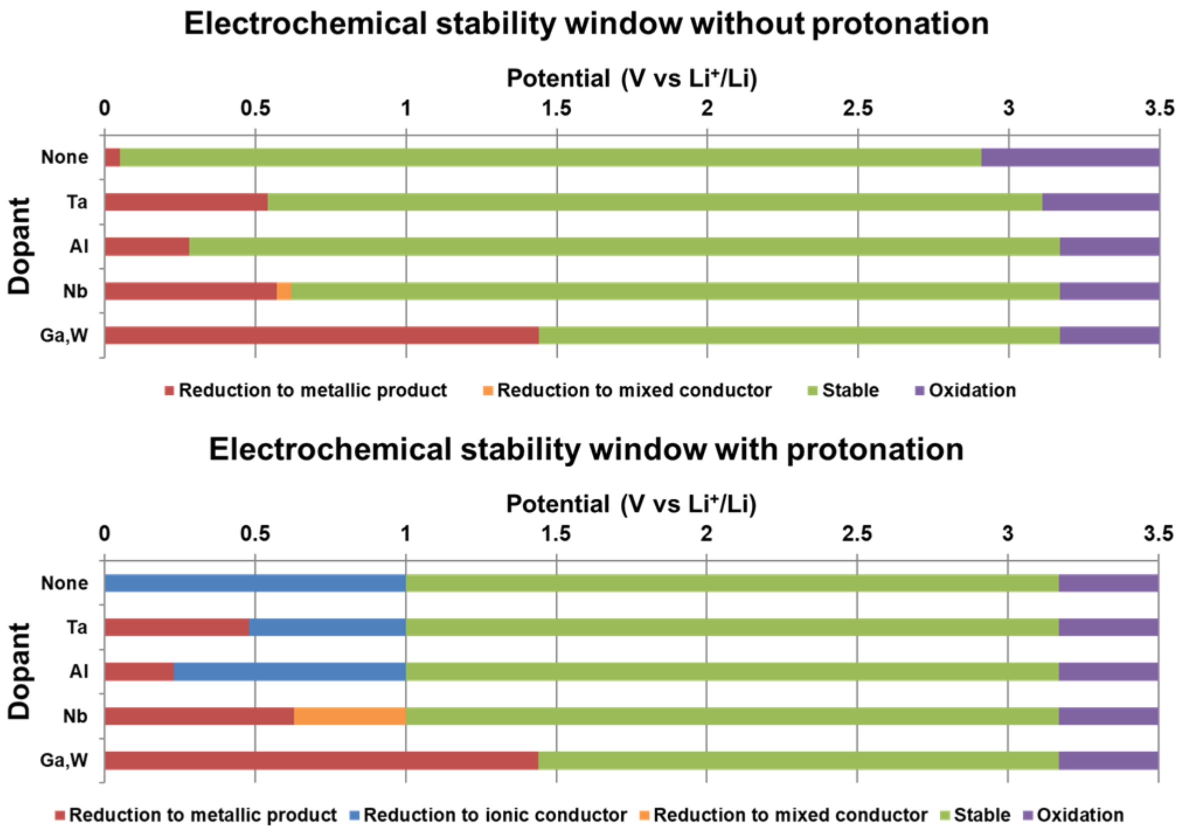

Supplementary Figure 13. Electrochemical stability window of all the samples under study, evaluated by DFT calculations. The colour scheme indicates the electrical properties of the decomposed products; red: metallic and electronically conductive, orange: electronically and ionically conductive, blue: ionically conductive but electronically insulating, and green: stable and does not decompose. When lithium ions are exchanged with protons for Ta- or Al-LLZO, electronically insulating products at the LLZO/lithium metal interface are produced.

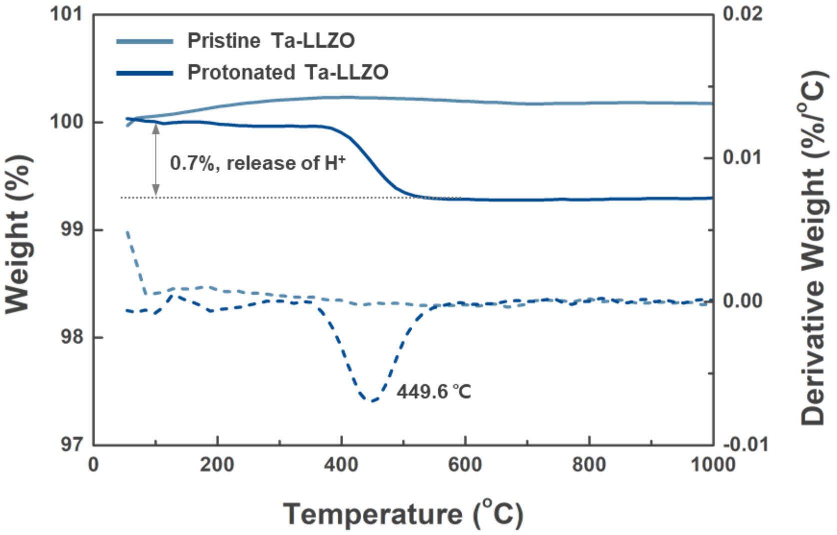

Supplementary Figure 14. Thermogravimetric analysis (TGA) curves of (a) pristine and (b) protonated Ta-LLZO pellets, revealing the partial substitution of Li^+ ions in LLZO with H^+. Data were recorded under an atmosphere of N_2.

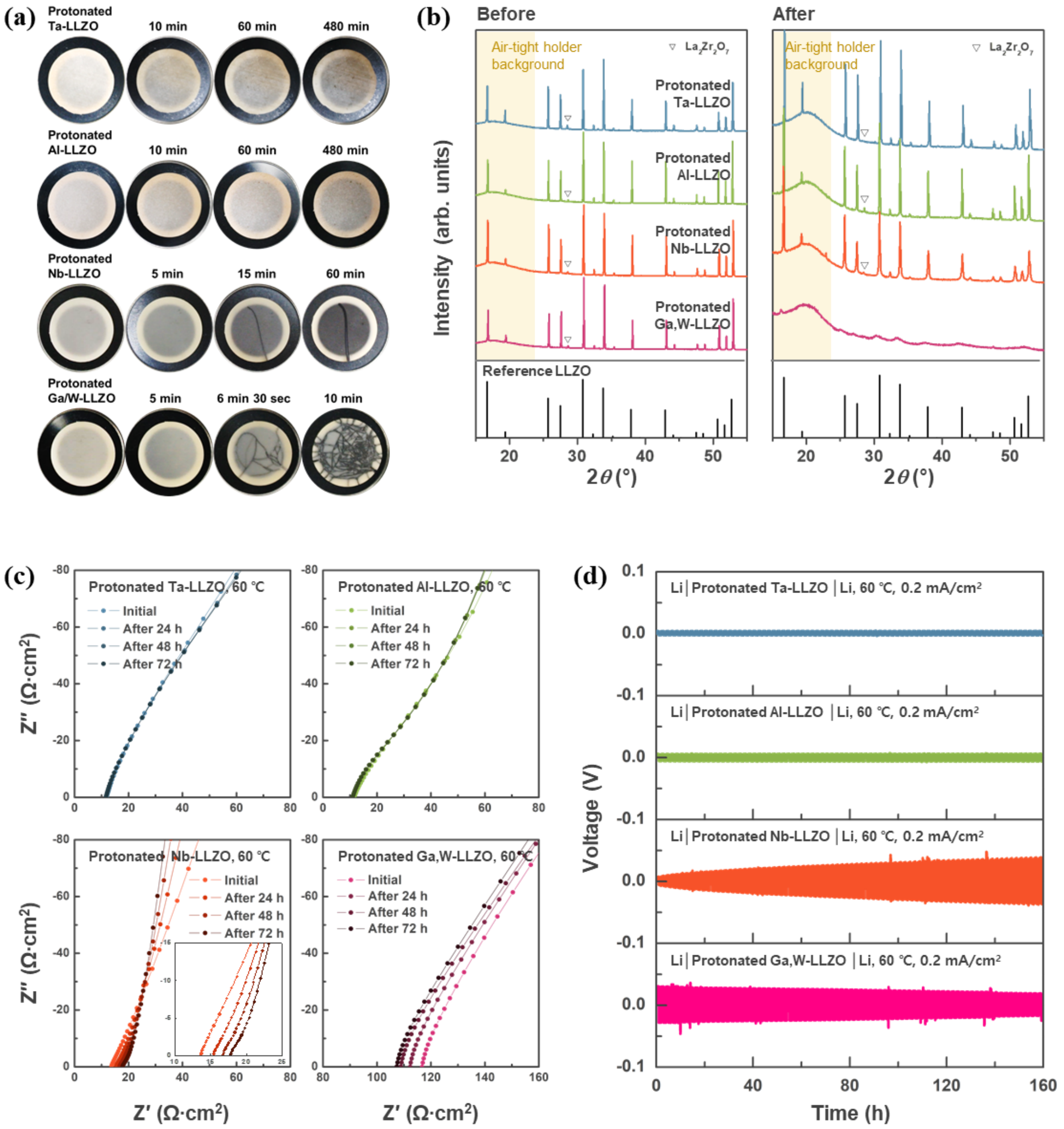

Supplementary Figure 15. Dopant stability of protonated LLZO against lithium metal. (a) Optical images (recorded over time) of protonated LLZO pellets in contact with lithium metal at 200 °C, after the system was assembled under a cold isostatic pressure of 250 MPa**.** (b) Comparison of XRD patterns of protonated LLZO pellets before (left) and after (right) contact with lithium metal. The reference XRD pattern is also indicated (ICSD 01-080-6142). The XRD patterns of the pellets were recorded before (left) and after (right) the pellets came in

contact with lithium metal, (c) Variation of the EIS profiles with time (Li/LLZO/Au cells measured at 60 °C), and (d) Results of galvanostatic cycling experiments conducted with Li/LLZO/Li symmetric cells at 60 °C (current density: 0.2 mA cm^{-2}).

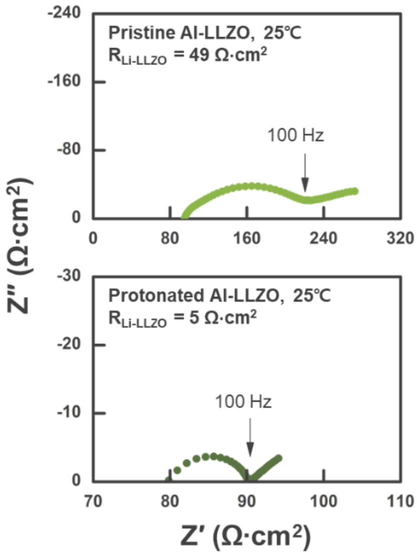

Supplementary Figure 16. Electrochemical impedance spectroscopy (EIS) results for Li/pristine Al-LLZO/Li (top) and Li/protonated Al-LLZO/Li (bottom) symmetric cells at 25 °C. The interfacial resistance exhibited by the Li/LLZO/Li symmetric cell decreased after the surface tailoring.

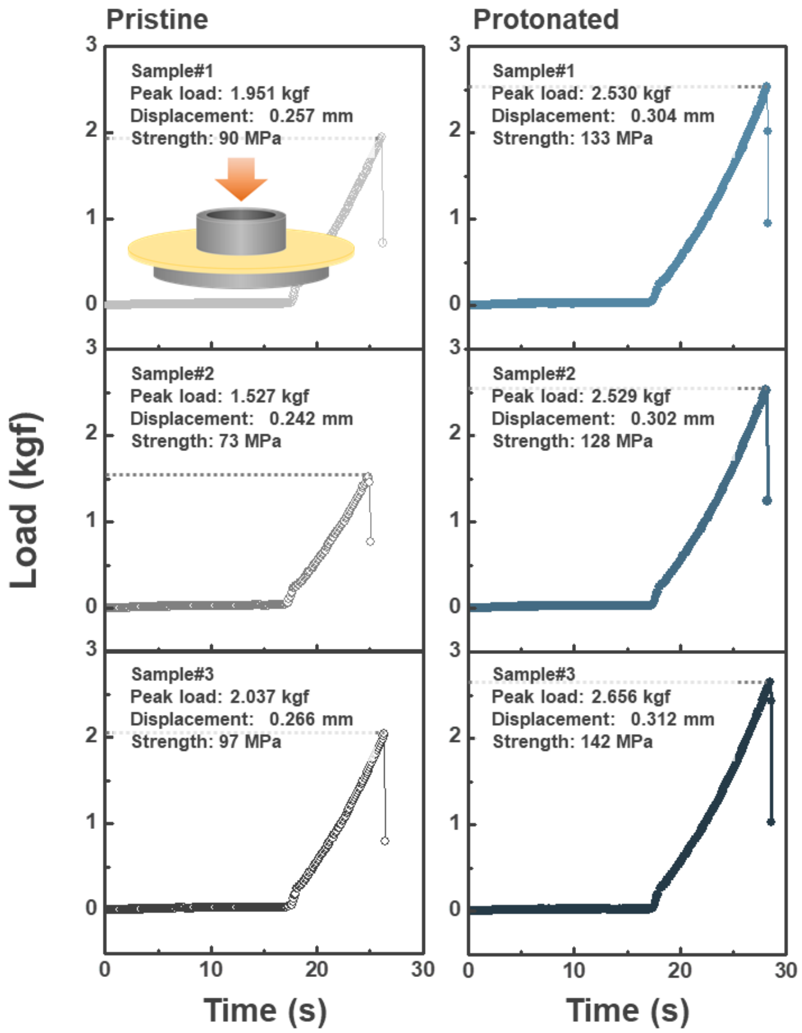

Supplementary Figure 17. The time-load curve of the pristine (left) and protonated Ta-doped LLZO (right) pellets exhibiting the maximum load that the pellet can endure during the ring-on-ring test. The tests were conducted by measuring the maximum value while applying a load at the same rates to the pellet placed between the two rings, as shown in the inset figure. The tensile strength of each pellet was calculated from the maximum load at which the pellet was broken, and the vertical displacement of the bent pellet. The protonated pellets present the larger maximum load value compared to the pristine pellets.

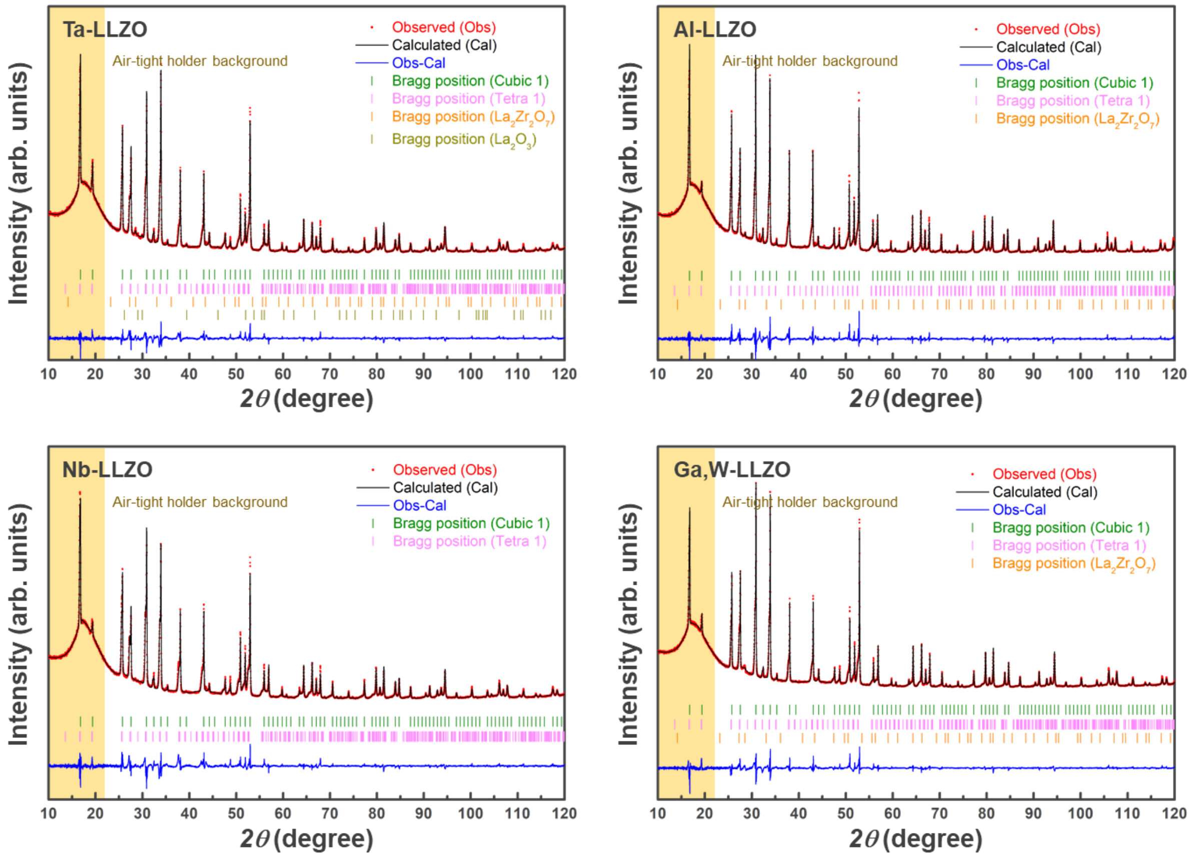

Sample	Phase	Lattice Parameters (*a*, Å)	Lattice Parameters (c, Å)	c/a	Cell vol. [$Å^3$]	Phase fraction (wt.%)	R_I (%)	R_F (%)
$Li_{6.5}La_3Zr_{1.5}Ta_{0.5}O_{12}$ R_p : 3.73 % R_{wp} : 5.42 %	Cubic	12.92980 (6)		1	2161.60 (2)	40.4 (7)	4.41	3.53
	Tetra	13.0347 (5)	12.9415 (9)	0.9928	2198.8 (2)	57.6 (12)	6.69	3.96
	$La_2Zr_2O_7$	10.8231 (6)		-	1267.8 (1)	1.62 (11)	-	-
	La_2O_3	3.9330 (7)	6.147 (3)	1.5629	82.34 (5)	0.36 (5)	-	-
$Li_{6.25}Al_{0.25}La_3Zr_2O_{12}$ R_p : 4.40 % R_{wp} : 6.54 %	Cubic	12.96374 (5)		1	2178.67 (14)	42.0 (7)	4.72	3.26
	Tetra	13.0483 (9)	13.015 (2)	0.9975	2216.0 (4)	57.1 (11)	6.94	4.56
	$La_2Zr_2O_7$	10.8035 (5)		-	1260.9 (1)	0.89 (9)	-	-
	La_2O_3			-	-	-	-	-
$Li_{6.5}La_3Zr_{1.5}Nb_{0.5}O_{12}$ R_p : 4.28 % R_{wp} : 6.38 %	Cubic	12.93028 (6)		1	2161.84 (2)	34.7 (6)	6.24	3.91
	Tetra	13.0420 (3)	12.9464 (9)	0.9927	2202.1 (2)	65.3 (9)	7.75	4.27
	$La_2Zr_2O_7$			-	-	-	-	-
	La_2O_3			-	-	-	-	-
$Li_{4.9}Ga_{0.5}La_3Zr_{1.7}W_{0.3}O_{12}$ R_p : 3.91 % R_{wp} : 5.70 %	Cubic	12.93939 (4)		1	2166.413 (11)	36.9 (5)	4.20	2.96
	Tetra	13.0267 (5)	12.9692 (11)	0.9956	2200.8 (2)	62.6 (9)	8.10	5.37
	$La_2Zr_2O_7$	10.83158		-	1270.8	0.55 (6)	-	-
	La_2O_3				-	-	-	-

Supplementary Figure 18. XRD patterns and Rietveld refinement results of the pristine LLZO pellets doped with various metals such as Ta, Al, Nb, and Ga,W. The refinement of the pellet specimen indicates that a significant amount of a tetragonal phase appears with small amounts of impurities such as $La_2Zr_2O_7$ and La_2O_3. The impurity phases present in the doped-LLZO samples are not critically specific to the composition of the LLZO, *i.e.* the dopant. Most importantly, the analysis revealed that the relative amount between the tetragonal phase and the cubic phase is comparable (Ta-LLZO: cubic 40.4 ± 0.7 %, tetragonal 57.6 ± 1.2 %, Al-LLZO: cubic 42.0 ± 0.7 %, tetragonal 57.1 ± 1.1 %, Nb-LLZO: cubic 34.7 ± 0.6 %, tetragonal 65.3 ± 0.9 %, Ga,W-LLZO: cubic 36.9 ± 0.5 %, tetragonal 62.6 ± 0.9 %). This ratio between the two phases in the pellet samples significantly differs from the results from the as-made powder XRD measurements (Supplementary Figure 2), which indicated the major cubic phase over 97%. It implies that the pressure-induced sintering process caused a partial phase transition from the cubic to the tetragonal phase. We highlight that the significant portion of the tetragonal phase is surface specific of the pellet sample and is attributed to the pellet geometry used in the XRD measurement. As shown in Supplementary Figure 19 and 20, we crushed and reground the pellet into the powder, and re-measured the powder XRD, which better represent the overall properties of the materials in the pellet samples. (The chemical composition of the corresponding pellets examined by the ICP-AES (Inductively Coupled Plasma-Atomic Emission Spectroscopy) is tabulated in Supplementary Table 1)

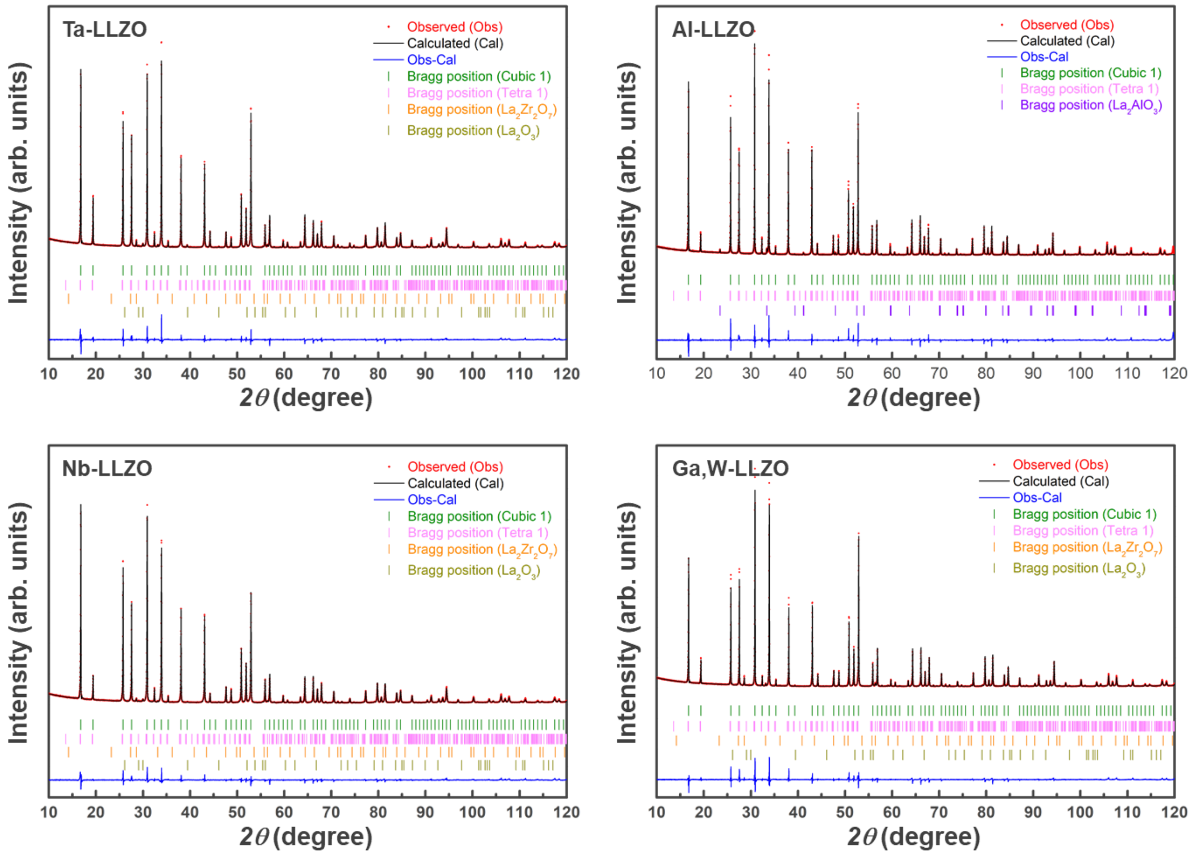

Sample	Phase	Lattice Parameters (*a*, Å)	Lattice Parameters (c, Å)	c/a	Cell vol. [Å^3]	Phase fraction (wt.%)	R_I (%)	R_F (%)
$Li_{6.5}La_3Zr_{1.5}Ta_{0.5}O_{12}$<br>$R_p$: 5.20 %<br>R_{wp} : 7.19 %	Cubic	12.93446 (5)		1	2163.94 (2)	94.8 (4)	3.05	3.30
	Tetra	13.03466	12.94145	0.9928	2198.784	3.48 (15)		
	$La_2Zr_2O_7$	10.80732			1262.275	1.38 (3)	-	-
	La_2O_3	3.93367	6.13873	1.5682	82.263	0.38 (2)	-	-
$Li_{6.25}Al_{0.25}La_3Zr_2O_{12}$<br>$R_p$: 5.88 %<br>R_{wp} : 8.09 %	Cubic	12.96765 (4)		1	2160.64 (1)	91.8 (4)	4.29	3.65
	Tetra	13.03466	12.94145	0.9928	2198.784	6.2 (2)		
	$LaAlO_3$	5.36569	13.11456	2.4442	326.991	2.00 (5)		
	La_2O_3							
$Li_{6.5}La_3Zr_{1.5}Nb_{0.5}O_{12}$<br>$R_p$: 4.88 %<br>R_{wp} : 6.72 %	Cubic	12.93433 (6)		1	2163.87 (2)	92.4 (4)	3.26	3.40
	Tetra	13.03466	12.94145	0.9928	2198.784	6.66 (14)		
	$La_2Zr_2O_7$	10.80732			1262.275	0.68 (3)		
	La_2O_3	3.93367	6.13873	1.5682	82.263	0.27 (2)		
$Li_{4.9}Ga_{0.5}La_3Zr_{1.7}W_{0.3}O_{12}$<br>$R_p$: 6.03 %<br>R_{wp} : 8.20 %	Cubic	12.94025 (4)		1	2166.85 (1)	92.7 (4)	3.86	3.83
	Tetra	13.03466	12.94145	0.9928	2198.784	4.9 (2)		
	$La_2Zr_2O_7$	10.80732			1262.275	2.48 (5)		
	La_2O_3							

* For the reliability of the data, only scale factor was set as a refinement parameter for the tetragonal phase and impurity phases.

Supplementary Figure 19. XRD patterns and Rietveld refinement results of the crushed/ground LLZO pellets in powder state, doped with various metals such as Ta, Al, Nb, and Ga,W. As shown in the Rietveld refinement results, each composition consists primarily of the cubic garnet phase and includes a small amount of the tetragonal phase and impure phases, such as $La_2Zr_2O_7$ (about 0.7~2.5% for all compositions) and La_2O_3 (less than 1% for Ta-LLZO and Nb-LLZO). We highlight again that the significant portion of the tetragonal phase is surface-specific of the pellet sample and is attributed to the pellet geometry (thick pellet) used in the XRD measurement. The detailed structural information of LLZOs and the relative phase fractions are similarly tabulated along with the original XRD patterns for the four doped-LLZO powder samples crushed from the pellets here. It indicates substantially higher contents of the cubic phase are indeed present in the pellets. As shown in the Rietveld refinement results, the cubic phase accounts for 94.8 wt% for Ta-LLZO, 91.8 wt% for Al-LLZO, 92.4 wt% for Nb-LLZO and 92.7 wt % for Ga,W-LLZO, respectively, which are significantly greater than the values determined from the pellet XRD data. It supports that the pelletized LLZOs are mainly composed of the cubic phase, however, the amount of the tetragonal phase was overestimated in the pellet XRD. Small amounts of the impure phases, such as $La_2Zr_2O_7$ (about 0.7~2.5% for all compositions) and La_2O_3 (less than 1% for Ta-LLZO and Nb-LLZO) were consistently observed in the powder samples.

** It should be noted that we did not apply the holder for the crushed pellet XRD measurement due to the empirical/practical reasons and for more accurate analysis. The main reason for using the air-tight holder was to protect the pellets, which were reused for another analysis or subsequent chemical/electrochemical tests, from the air exposure. Therefore, it was not critically necessary to apply the air-tight holder for powder samples that were not subjected to

the further analysis or experiments. Further, since the amount of the crushed pellet was small (about 0.2-0.3 g), it was practically difficult to adjust the z-axis with the air-tight holder so that the conventional holder for powder XRD was applied for more accurate analysis. Although there might be potential effects from air exposure of powder with increased surface area, it can be considered negligible in interpreting the results. The primary phenomenon that occurs when LLZO is exposed to air is the formation of Li_2CO_3 on the surface as reported by Cheng *et al*.[18] If there had been a significant reaction during the XRD measurement to cause the change in the LLZO structure, the peaks arising from Li_2CO_3 formation should have been observed. However, it was not observed in our results of the crushed pellets. Thus, the possibility of the massive Li_2CO_3 formation during XRD measurement as much as to affect the LLZO phase analysis can be excluded. For these reasons, we have concluded that the displayed results (obtained from XRD measurement without air-tight holder) were still reliable when considering the original purpose of the XRD analysis on the crushed pellets, the proof of the tetragonal LLZO phase that exists only locally on the pellet surface.

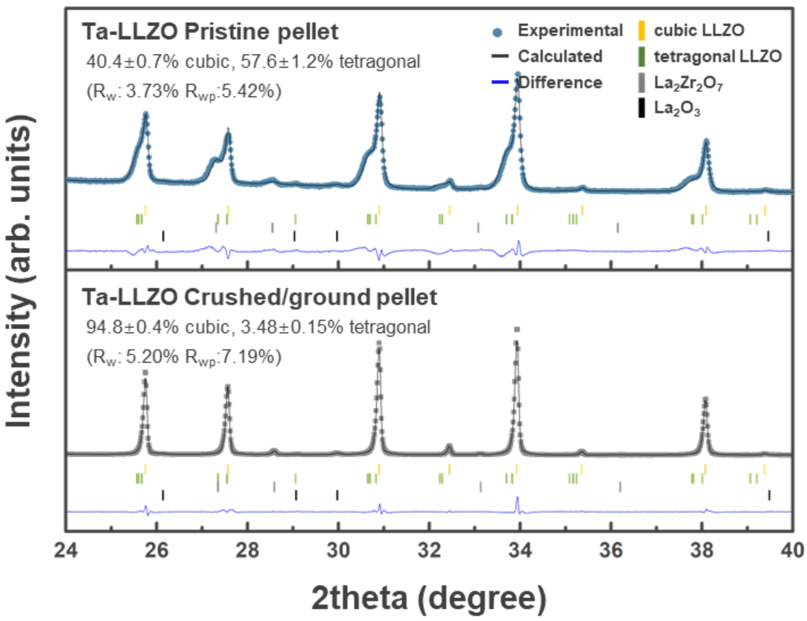

Supplementary Figure 20. XRD patterns recorded for a pristine pellet (blue line) and the corresponding crushed/ground pellet in the powder state (black line) of Ta-LLZO. We simply compared the XRD peaks of the pellet sample with those from the powder sample obtained by crushing/grinding the pellet for the Ta-LLZO. It evidently presents that the peaks from the tetragonal phase are hardly detected in the powder sample crushed from the pellet. We note that this discrepancy arises because the surface of the pellets contains relatively more tetragonal phase than the bulk, which is derived from the pellet preparation process. It is widely known that the formation of the tetragonal phase can be locally promoted during the manufacturing process of the pellets, which involves the application of pressure (during the sintering process) and polishing to remove the surface contamination such as Li_2CO_3 or LiOH[11,15,19]. During the preparation of our thick LLZO pellet, the surface of the pellet gets exposed to this condition more vulnerably, inducing the preferred formation of the tetragonal phase at the surface. When considering an X-ray penetration depth of~10–50 μm[20,21] , the XRD results on the 300–350-μm-thick pristine pellets would preferably represent the surface region of the thick samples and cannot be regarded as showing the overall bulk property of the pellet.

Thus, it is reasonable to consider that the coexistence of the two phases with the significant portion of the tetragonal phase is a characteristic that is limited to the surface.

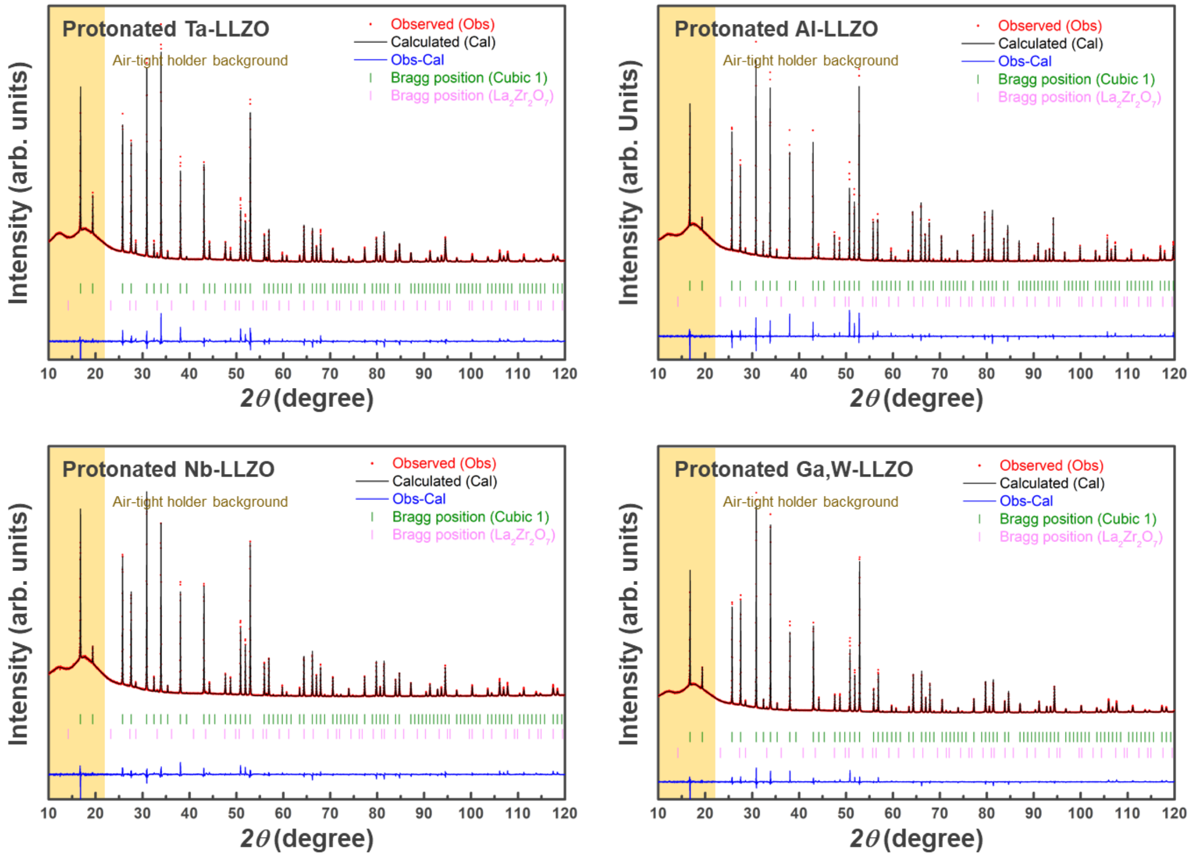

Sample	Phase	Lattice parameter (a, Å)	Cell vol.[Å^3]	Phase fraction (wt.%)	R_I(%)	R_F(%)
H-$Li_{6.5}La_3Zr_{1.5}Ta_{0.5}O_{12}$<br>$R_p$: 4.42 %<br>R_{wp} : 6.99 %	Cubic	12.92955 (3)	2161.473 (9)	97.2 (5)	5.22	4.17
	$La_2Zr_2O_7$	10.81167 (17)	1263.80 (3)	2.81 (6)	-	-
H-$Li_{6.25}Al_{0.25}La_3Zr_2O_{12}$<br>$R_p$: 3.75 %<br>R_{wp} : 6.08 %	Cubic	12.96768 (2)	2180.656 (7)	98.1 (4)	6.19	3.91
	$La_2Zr_2O_7$	10.8119 (3)	1263.89 (7)	1.88 (8)	-	-
H-$Li_{6.5}La_3Zr_{1.5}Nb_{0.5}O_{12}$<br>$R_p$: 3.85 %<br>R_{wp} : 6.15 %	Cubic	12.93253 (3)	2162.971 (9)	98.7 (4)	5.08	4.14
	$La_2Zr_2O_7$	10.8110 (3)	1263.56 (7)	1.26 (8)	-	-
H-$Li_{4.9}Ga_{0.5}La_3Zr_{1.7}W_{0.3}O_{12}$<br>$R_p$: 3.42 %<br>R_{wp} : 5.15 %	Cubic	12.94367 (2)	2168.562 (7)	98.0 (3)	4.49	3.42
	$La_2Zr_2O_7$	10.8095 (3)	1263.05 (5)	1.97 (8)	-	-

Supplementary Figure 21. XRD patterns and Rietveld refinement results of the protonated LLZO pellets doped with various metals such as Ta, Al, Nb, and Ga,W. Each pellet exhibited a cubic garnet phase containing an impurity phase ($La_2Zr_2O_7$).

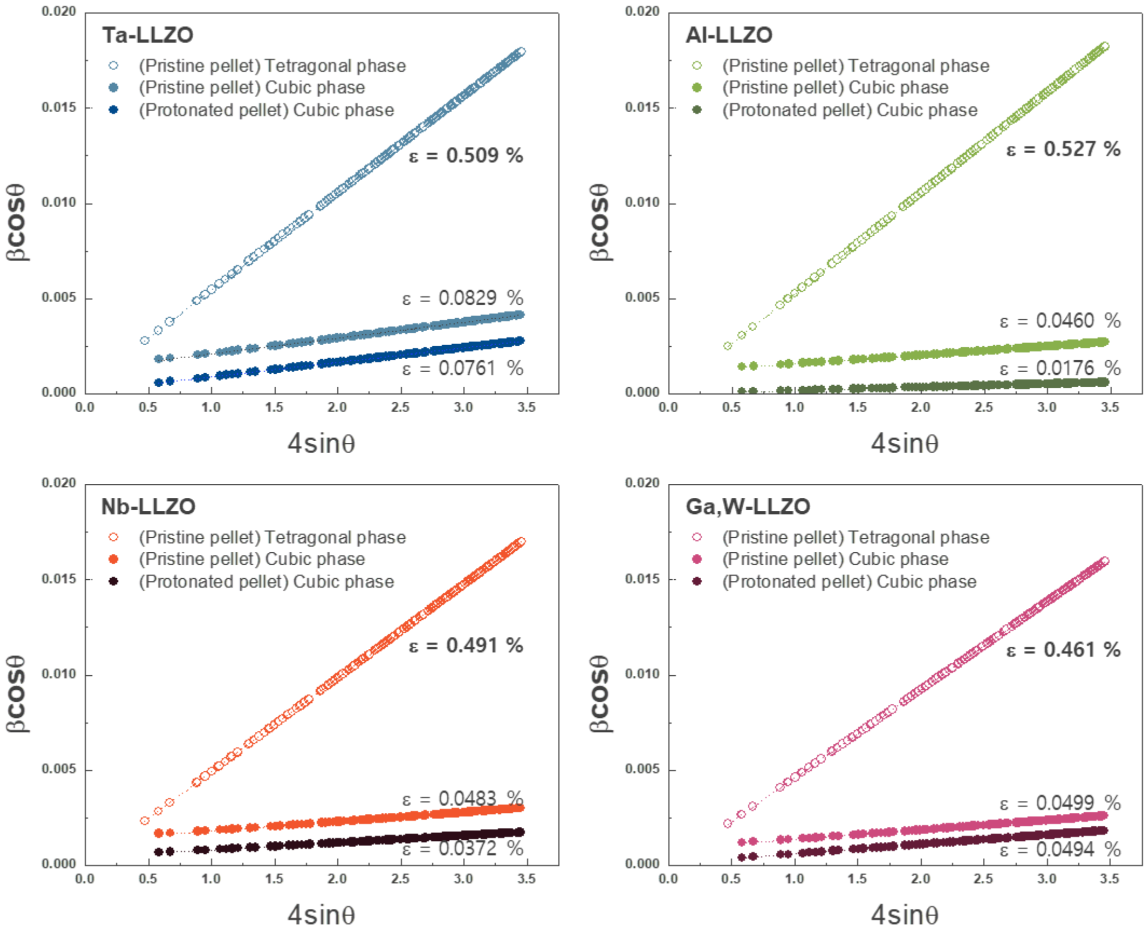

Supplementary Figure 22. Size–strain plots for LLZO pellets before and after surface tailoring. The graphs were plotted based on the Williamson–Hall analysis and Rietveld refinement results presented in Supplementary Figure 18 and 21. Williamson–Hall plots were obtained using the equation, $\beta_{hkl} \cdot cos\theta = \frac{K\lambda}{D} + 4\varepsilon \cdot sin\theta$ with the integral breadth that was calculated from the U, X, and Y values from the Rietveld refinement process. The micro-strain values were derived from the slope of the linear fit. Tetragonal phases in the pristine garnet samples exhibited micro-strain values (0.46 – 0.53 %) that were higher than the micro-strain values exhibited by the cubic phase (0.046 – 0.083 %). After the protonation, the micro-strain was released accompanied by the disappearance of the tetragonal phase. Protonated cubic phase also showed decrease of the micro-strain compared to the pristine cubic phase.

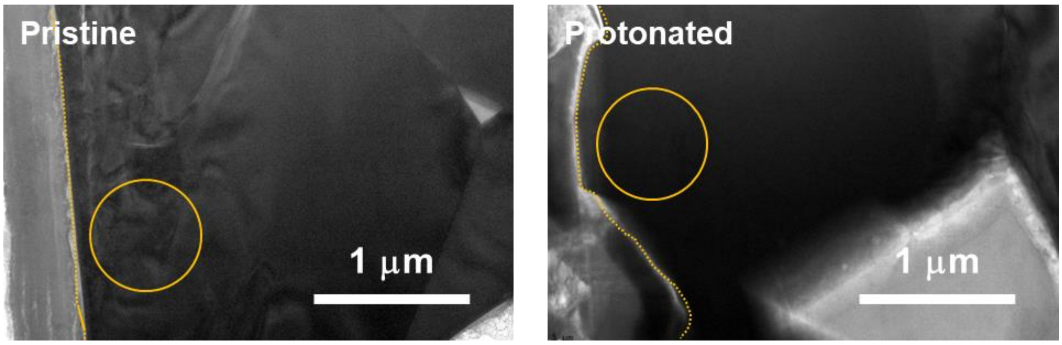

Supplementary Figure 23. Bright-field transmission electron microscopy (TEM) images of the surface of a Ta-LLZO pellet before and after surface tailoring. The wave pattern in the pristine pellet indicates the existence of a strain field.

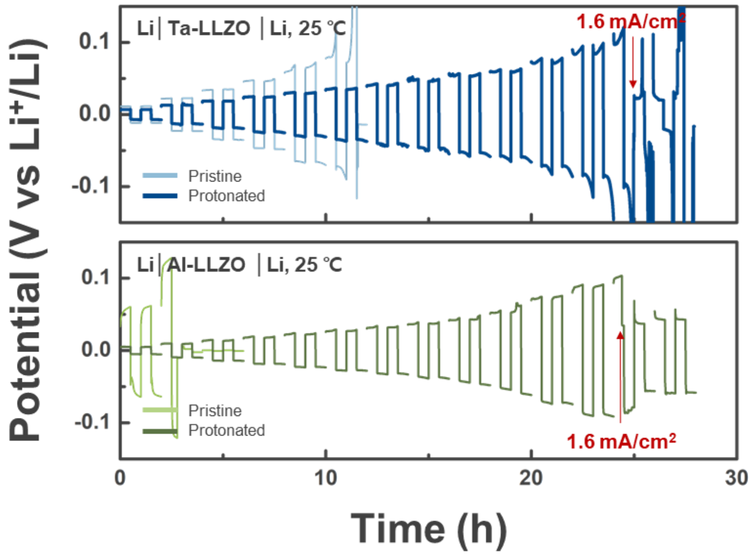

Supplementary Figure 24. Critical current densities of pristine (light blue and green lines) and surface-tailored (dark blue and green lines) Ta- or Al-LLZO as determined by the galvanostatic cycle tests on the symmetrical cells at 25 °C. The current density was increased in two steps (0.1 mA cm^{-2} to 1 mA cm^{-2}, step size: 0.1 mA cm^{-2}; from 1.0 mA cm^{-2} to 2.0 mA cm^{-2}, step size: 0.2 mA cm^{-2}). The cells were fabricated using the LLZO pellet (diameter: 14 mm) and 100 um-thick lithium metal on 10-μm-thick copper foil (area: 0.5 cm^{2}). They were cycled twice over 30 minutes to achieve lithium plating/stripping (at each current density). The cells fabricated using protonated Ta-LLZO and protonated Al-LLZO exhibited the critical current densities (approximately 1.6 mA cm^{-2}), which were remarkably higher than those exhibited by the pristine Ta- and Al-LLZOs.

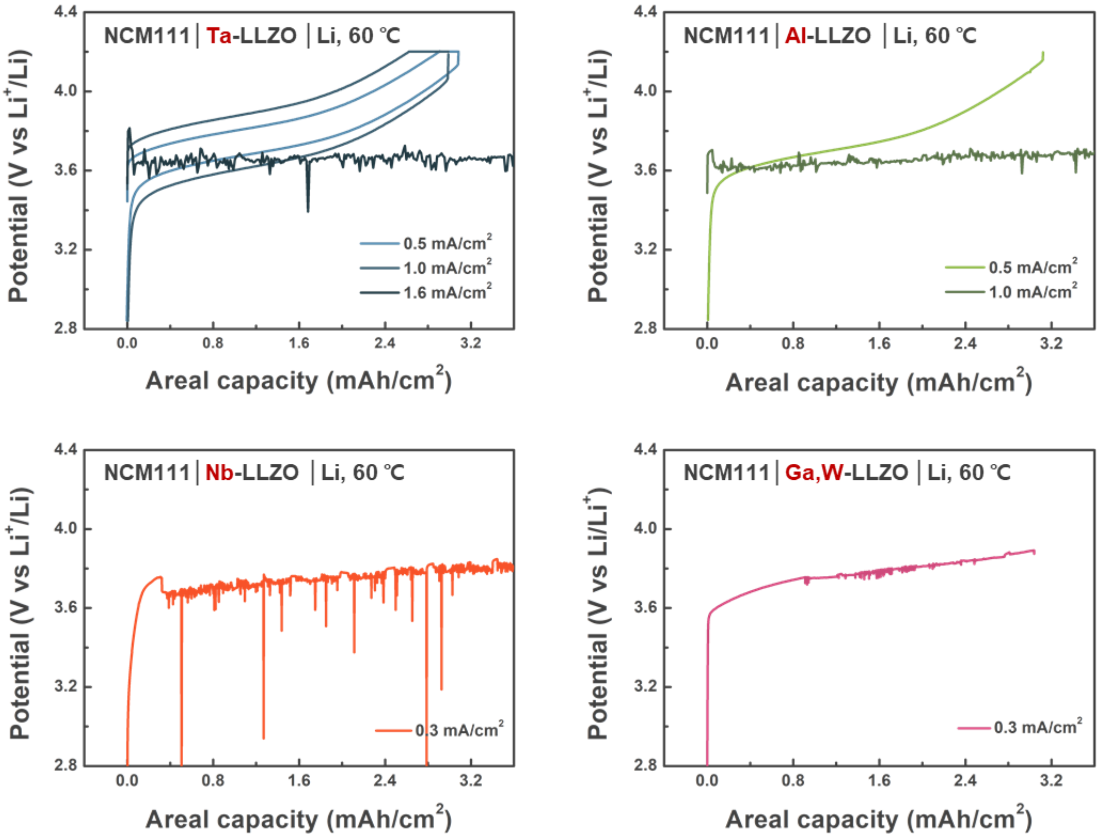

Supplementary Figure 25. Electrochemical performances of hybrid solid-state full cells measured at 60 °C, employing a lithium metal anode and a pristine LLZO with various dopants. The extent of operating current density increase was more in the case when Ta- and Al-LLZO were used than the case when Nb- and Ga,W-LLZO were used. The maximum increase in the operating current density was observed when the surface-tailored Ta- and Al-LLZO were used (Figure 3b).

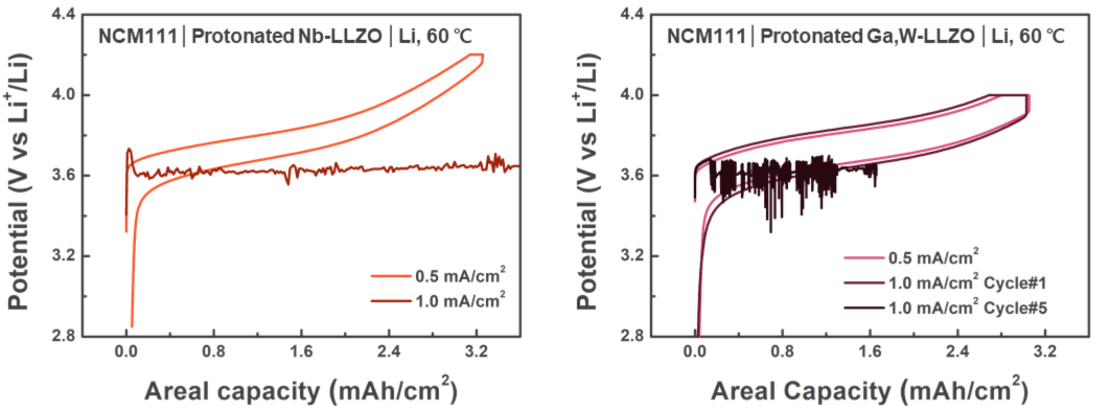

Supplementary Figure 26. Electrochemical performances of hybrid solid-state full cells measured at 60 °C. The cells were fabricated using a lithium metal anode and a protonated LLZO doped with Nb- and Ga,W. It should be noted that the acid treatment process did not substantially mitigate lithium propagation through the Nb- and Ga,W-LLZO electrolytes. The results revealed the importance of controlling the nature of the by-products at the interface.

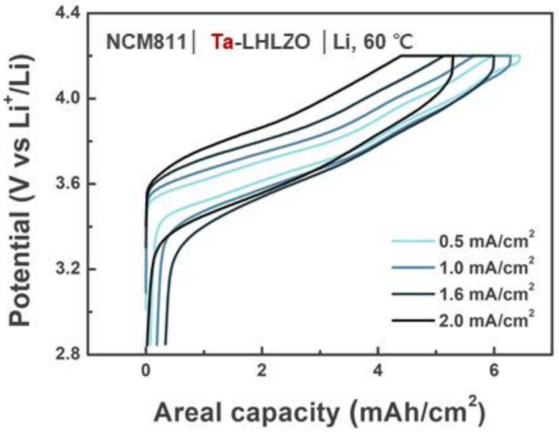

Supplementary Figure 27. Electrochemical performances of hybrid solid-state full cells at 60 °C, fabricated using a lithium metal anode, surface-stabilised Ta-LLZO, and NCM811cathode exhibiting a high capacity of 6.4 mAh cm^{-2}.

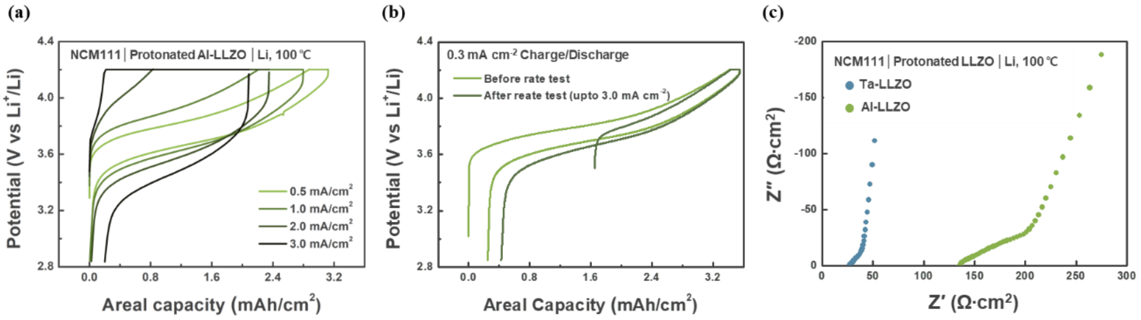

Supplementary Figure 28. (a–b) Voltage profiles of Li/protonated Al-LLZO/NCM111 cells at 100 °C under the current densities of (a) 0.5, 1.0, 2.0, and 3.0 mA cm^{-2} and (b) 0.3 mA cm^{-2} before and after the tests (shown in (a)). (c) Initial EIS profiles recorded with NCM111/protonated Ta- or Al-LLZO/Li hybrid cells at 100 °C before the electrochemical tests were conducted (Figure 3e). At the current densities greater than 4 mA/cm^2, the cell employing Al-LLZO delivers far smaller capacity than Ta-LLZO cells. We believe that the primary reason for the inferior rate capability of the Al-LLZO cell is the comparatively slower migration of lithium in the Al-LLZO than that of the Ta-LLZO. When we performed EIS on the full cells at 100 °C before cycling, it was found that Al-LLZO cell displayed a larger impedance than that of the Ta-LLZO. The intrinsically large impedance of the cell would be consequently accompanied by a large over-potential at higher current densities and the subsequent reduction in the capacity. It is also noteworthy that the reduction in the capacity at high current densities is not ascribed to an irreversible degradation of the cell such as deterioration of the Al-LLZO. It is evident here that, even after the cycling at high current densities, the capacity could be recovered when the cell is cycled again at a low current density of 0.3 mA cm^{-2}.

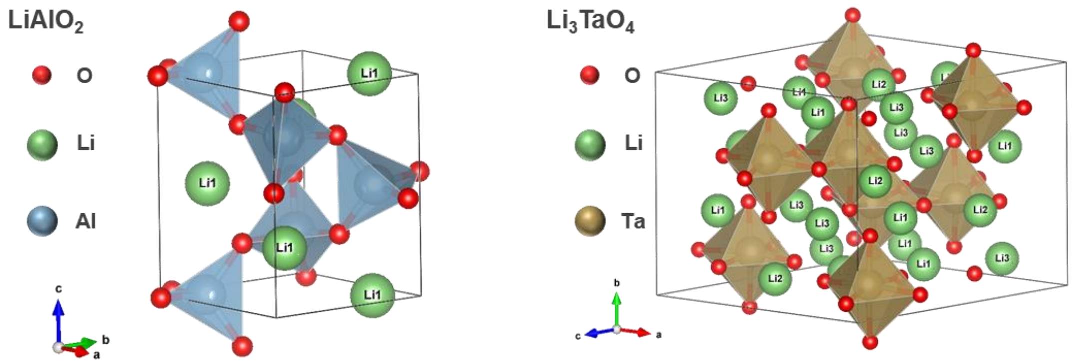

Supplementary Figure 29. Crystal structures of lithium aluminium oxide (γ-$LiAlO_2$) and lithium orthotantalate (β-Li_3TaO_4). The lithium sites are marked with numbers in each structure. We acknowledge that the reaction products of protonated Ta- or Al-LLZO with the lithium metal may affect the rate capability of the cells. In this respect, we further investigated the ionic conductivities of Li_3TaO_4 and $LiAlO_2$, which are the reactant products of protonated Ta- and Al-LLZO with lithium metal. Lithium-ion diffusion barriers were calculated using the nudged elastic band (NEB) method and implemented in the Vienna ab initio simulation package (VASP), by considering all possible pathways of the lithium vacancy migration in the crystal structure. In $LiAlO_2$, there is only one diffusion path between the Li1 sites. In Li_3TaO_4, there are, in total, 14 paths for lithium diffusion to occur (three different sites: Li1, Li2, and Li3). The energy barriers for lithium migration through Li1–Li2 (0.17–0.29 eV) and Li2–Li3 (0.29–0.39 eV) were extremely low. These energy barriers were significantly lower than the barrier observed in $LiAlO_2$ (0.52 eV, Supplementary Table 4). The energy barrier values in brackets in the table refer to the barrier in its reverse direction, owing to the asymmetric diffusion path from one site to the other. A large Li-ion migration barrier in $LiAlO_2$ was also reported by Wiedemann, D. *et al.*[22] The results revealed that the interfacial decomposition product

produced from protonated Al-LLZO exhibited lower ionic conductivity (compared with the case of protonated Ta-LLZO). It also implies that the different nature of the reaction products with respect to the lithium migration would contribute to the difference in the overall rate performance of the two LLZOs.

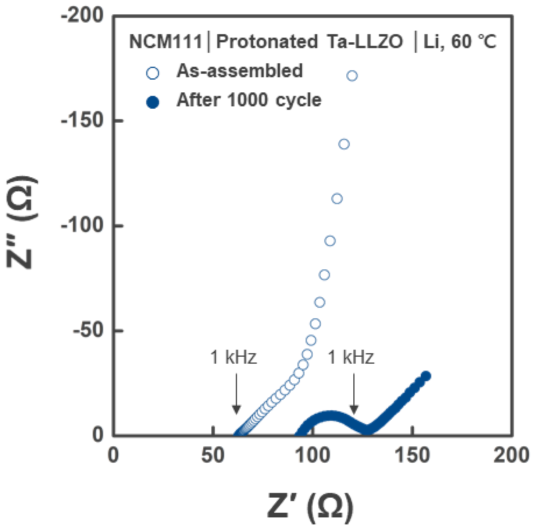

Supplementary Figure 30. EIS profiles of NCM111/protonated Ta-LLZO/Li hybrid cells at 60 °C before and after cycling, as shown in Figure 4a. The change in the cell impedance after 1000 cycles is remarkably small, considering the EIS spectrum exhibits signals corresponding to all the components and interfaces of the cell. It is noted that numerous physical or (electro) chemical reactions can potentially influence the EIS spectra (such as Solid Electrolyte Interphase (SEI) growth/decomposition, active material degradation/exfoliation, or loss of electric contact[23-26]).

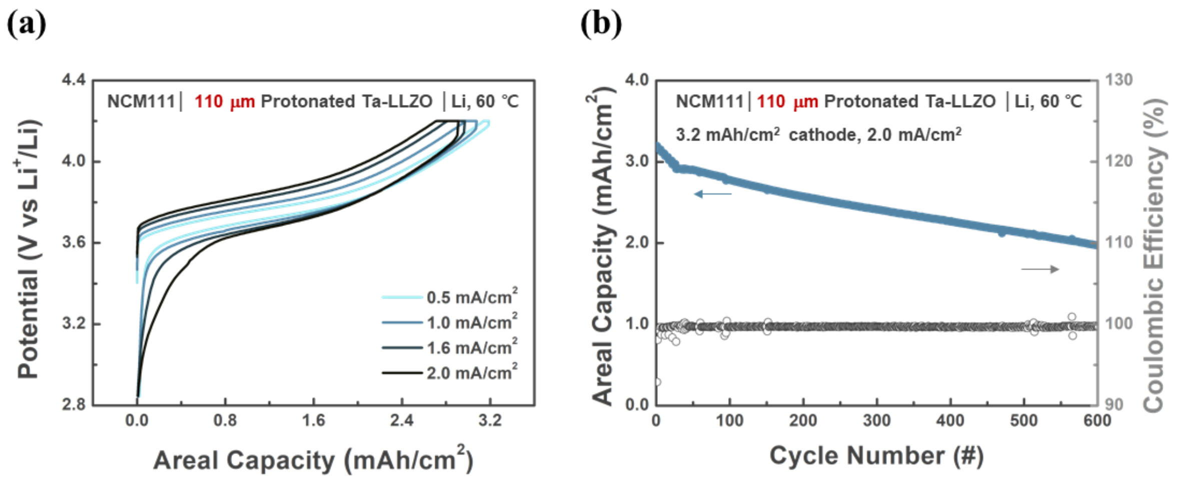

Supplementary Figure 31. Electrochemical performance of hybrid solid-state full cells at 60 °C, fabricated using a lithium metal anode and thin 110 μm thick surface-stabilised Ta-LLZO. (a) Voltage profiles of the Li/LLZO/NCM111 cell and (b) Cyclability at 60 °C. The cells could be operated for a prolonged time (600 cycles) without short-circuiting under a high current density of 2 mA cm^{-2}. A high capacity of 3.2 mAh cm^{-2} was recorded. Long-term cycle stability (> 600 cycles) was exhibited by the protonated 110 μm thick Ta-LLZO. Although this cell exhibited less capacity than the cell fabricated using a thicker pellet, lithium metal was plated/stripped during the long-term cycling process in a stable manner, owing to the to the enhanced interfacial stability achieved through surface tailoring. The discrepancy in the capacity retentions between the tape-cast and hot-pressed pellet cells can be potentially attributed to the degradation of the Li/LLZO interface. The degradation can be attributed to the differences in their surface properties (such as the composition and microstructure), as described in Supplementary Figure 32.

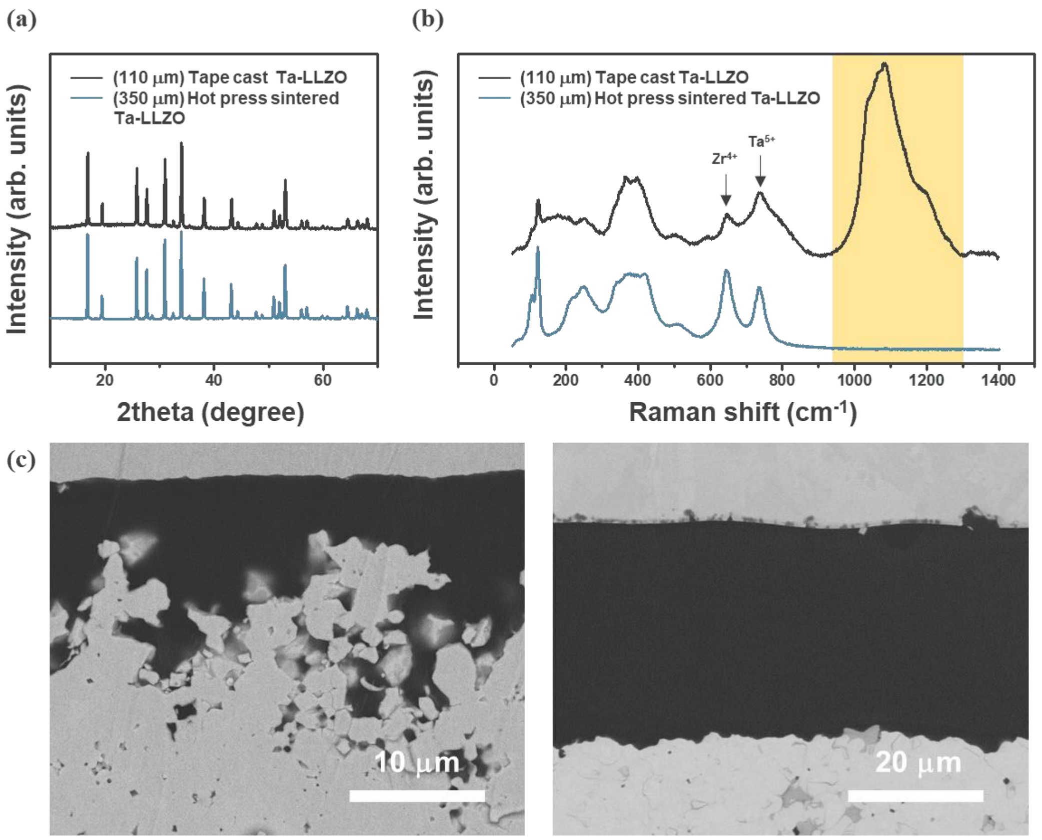

Supplementary Figure 32. The comparisons of physical properties of hot press-sintered and tape cast Ta-LLZO electrolytes. (a) XRD patterns of the hot-pressed and sintered Ta-LLZO (thickness: 350 μm) and tape-cast Ta-LLZO (thickness:110 μm) electrolyte surfaces after protonation. The electrolytes exhibited cubic-garnet-like structures containing 1–2% of $La_2Zr_2O_7$. The results implied that the crystal structure of the electrolytes did not significantly differ from each other. (b) Raman spectral profiles of the hot-pressed and sintered Ta-LLZO (thickness: 350 μm) and tape-cast Ta-LLZO (thickness:110 μm) electrolyte surface after protonation. Several bands were observed in the spectral profile of the tape-cast electrolyte in the range of 950 to 1300 cm^{-1} (indicated by yellow range). These bands were absent in the spectral profiles of the 350 μm-thick pellet. While the origin of these bands is not clear, we hypothesised that these could be attributed to an amorphous phase or the residual carbon compounds present on the electrolyte surface. The residual carbon compounds originated from the organic binders used in the tape-cast process conducted under different sintering conditions.

Raman spectra were collected using a Renishaw inVia^{TM} Raman microscope with a 514 nm laser, 2400 lines/mm holographic grating, and 50× magnification. In order to prevent the exposure of the electrolytes to the ambient atmosphere, all the samples were analysed in the sealed state using ketone tape. (c) Cross-sectional SEM images of surface tailored pellet and tape-cast electrolyte in contact with the lithium metal. The tape-cast electrolyte exhibited a less rough and porous surface morphology. The time for surface tailoring was shortened to 10 minutes, taking into account that the thickness of tape-cast electrolyte is about 1/3 of pellets and the average grain size of tape-cast electrolyte is smaller than that of the pellet. This could lead to the difference in the corrosion effect, forming a less rough and porous surface.

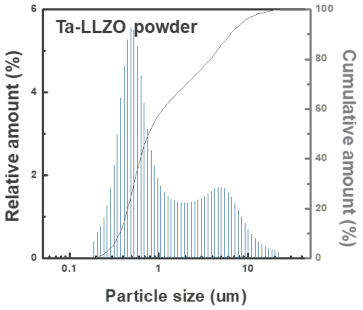

Cumulative amount (%)	Size (um)
10	0.344
20	0.427
30	0.505
40	0.604
50	0.782
60	1.256
70	2.395
80	4.28
90	7.02
95	10.37

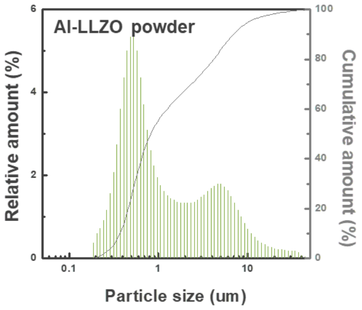

Cumulative amount (%)	Size (um)
10	0.342
20	0.423
30	0.499
40	0.592
50	0.750
60	1.146
70	2.136
80	3.83
90	6.35
95	9.42

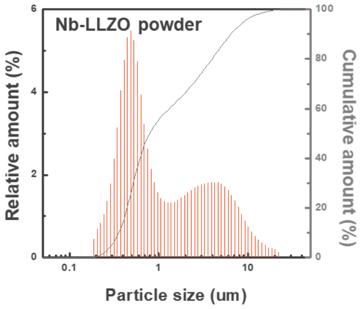

Cumulative amount (%)	Size (um)
10	0.344
20	0.427
30	0.507
40	0.610
50	0.800
60	1.298
70	2.481
80	4.25
90	6.71
95	9.31

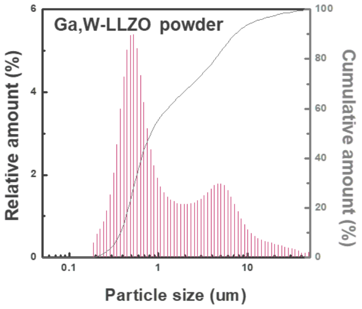

Cumulative amount (%)	Size (um)
10	0.338
20	0.414
30	0.483
40	0.566
50	0.696
60	0.997
70	1.823
80	3.60
90	6.84
95	12.11

Supplementary Figure 33. Particle size distribution of the LLZO powders with various dopants, Ta, Al, Nb and Ga,W. All the samples exhibit similar bimodal particle size distribution with peaks at 0.4 and 4 µm, showing roughly the same average particle sizes with D50 ~ 0.7~0.8 um.

Supplementary Table 1. Chemical composition of LLZO pellets from ICP-AES results. Atomic ratio of Li:La:Zr:dopant is normalized by the La content in the formula $Li_{7-x}La_3Zr_{2-y}(Dopant)_aO_{12}$.. It indicates that the final compositions of the pellets well matched with the target compositions. Along with the fact that the R factors of the refinement (Supplementary Figure 18 and 21) are relatively low when applied to the nominal composition, it supports that the stoichiometry of each composition does not deviate from the designed target compositions.

Elements	Nominal composition	ICP-AES result	
		Pristine	Protonated
Li : La : Zr : Ta	6.5 : 3 : 1.5 : 0.5	6.58 : 3 : 1.50 : 0.52	6.54 : 3 : 1.49 : 0.52
Li : La : Zr : Al	6.25 : 3 : 2 : 0.25	6.35 : 3 : 2.05 : 0.34	6.31 : 3 : 2.02 : 0.34
Li : La : Zr : Nb	6.5 : 3 : 1.5 : 0.5	6.66 : 3 : 1.48 : 0.52	6.54 : 3 : 1.46 : 0.53
Li : La : Zr : Ga : W	4.9 : 3 : 1.7 : 0.5 : 0.3	5.08 : 3 : 1.74 : 0.47 : 0.32	4.99 : 3 : 1.72 : 0.46 : 0.31

*0.01 g of each crushed pellet was dissolved in a 3/0.4/100 (v/v/v) mixture of 37 wt% hydrochloric acid, 70 wt% nitric acid, and deionized water at 170 °C. As prepared solution was used for the element analysis, except for Li and Zr analysis that used further diluted solution to 1/20 of the original concentration by a 2/100 (v/v) dilution of nitric acid. ICPS-8100 (Shimadzu, Japan) was used for the measurement and the software provided by the manufacturer of the instrument was used for data acquisition

Supplementary Table 2. Phase equilibria of LLZO doped with various metals at the potential for the first reduction.

Composition	Potential (V vs Li^+/Li)	Phase equilibria
$Li_7La_3Zr_2O_{12}$	0.05	Li_2O, Zr_3O, La_2O_3
$Li_{6.5}La_3Zr_{1.5}Ta_{0.5}O_{12}$	0.54	$Li_6Zr_2O_7$, La_2O_3, Li_5TaO_5, Ta
$Li_{6.25}Al_{0.25}La_3Zr_2O_{12}$	0.28	$Li_6Zr_2O_7$, La_2O_3, Li_5AlO_2, $ZrAl_2$
$Li_{6.5}La_3Zr_{1.5}Nb_{0.5}O_{12}$	0.62	$Li_6Zr_2O_7$, La_2O_3, Li_2O, $LiNbO_2$
$Li_{4.9}Ga_{0.5}La_3Zr_{1.7}W_{0.3}O_{12}$	1.44	$Li_6Zr_2O_7$, La_2O_3, $LiGaO_2$, $Li4WO_5$, W

Supplementary Table 3. Phase equilibria of protonated LLZO doped with various metals at the potential for the first reduction.

Composition	Potential (V vs Li^+/Li)	Phase equilibria
$Li_6HLa_3Zr_2O_{12}$	1.00	LiOH, $Li_6Zr_2O_7$, $La(OH)_3$, La_2O_3
$Li_{4.5}H_2La_3Zr_{1.5}Ta_{0.5}O_{12}$	1.00	$Li_6Zr_2O_7$, La_2O_3, $La(OH)_3$, LiOH, Li_3TaO_4
$Li_{4.25}H_2Al_{0.25}La_3Zr_2O_{12}$	1.00	$Li_6Zr_2O_7$, $La_2Zr_2O_7$, La_2O_3, LiOH, $LiAlO_2$
$Li_{4.5}H_2La_3Zr_{1.5}Nb_{0.5}O_{12}$	1.00	$Li_6Zr_2O_7$, La_2O_3, $La(OH)_3$, LiOH, $Li_8Nb_2O_9$
$Li_4Ga_{0.5}HLa_3Zr_{1.7}W_{0.3}O_{12}$	1.44	$Li_6Zr_2O_7$, $La_2Zr_2O_7$, La_2O_3, LiOH, $LiGaO_2$, W

Supplementary Table 4. Lithium-ion diffusion energy barrier in lithium aluminium oxide (γ-$LiAlO_2$) and lithium orthotantalate (β-Li_3TaO_4) calculated using the nudged elastic band (NEB) method implemented in VASP. All possible pathways of lithium vacancy migration in the crystal structure were considered, corresponding to the sites shown in Supplementary Figure 29.

	**Ionic migration path**		**Migration energy barrier (eV)**
**$LiAlO_2$**	Li1 ↔ Li1		0.52
**Li_3TaO_4**	Li1 ↔ Li1	Path1	0.58
		Path2	0.60
	Li2 ↔ Li2	Path1	0.41
		Path2	0.47
	Li3 ↔ Li3	Path1	0.17
		Path2	1.15
	Li1 ↔ Li2	Path1	0.29 (0.17)
		Path2	0.43 (0.32)
		Path3	0.54 (0.42)
	Li2 ↔ Li3	Path1	0.39 (0.29)
		Path2	0.50 (0.40)
		Path3	0.55 (0.45)
	Li3 ↔ Li1	Path1	0.37 (0.40)
		Path2	0.78 (0.81)

Supplementary Table 5. Parameters considered for estimating the energy densities of the full cells.

	Composition	NCM111	NCM811
**Cathode**	Areal capacity (mAh cm^{-2})	3.2	6.0
	Thickness (mm)	66	81
**Solid electrolyte**	Thickness (mm)	110	330
**Anode**	Initial Li thickness (mm)	20	20
	Plated Li thickness (mm)	16	31
**Current collector**	Al for cathode (mm)	12	12
	Cu for anode (mm)	10	10
**Total thickness (mm)**		234	484
**Cell area (cm^2)**		30.2	30.2
**Cell volume including package film (L)**		0.780	1.609
**Discharge V_{ave} (V)**		3.79	3.8
**Energy density (Wh L^{-1})**		470	428

Supplementary Table 6. Summary of the electrochemical plating/stripping performances of the cells (garnet electrolyte and lithium metal) reported in the literatures.

Data point	Cell configuration	Plating current density (mA/cm^2)	Per-cycle plating capacity (mAh/cm^2)	No. of cycles	Cumulative lithium metal capacity (mAh/cm^2)	References
S1	Li|LLZT-RAT|LCO	0.022	0.220	100	22.0	Nano Energy 61, 119 (2019)
	Li|LLZT-RAT|LFP	0.029	0.286	150	42.9	
	Li|LLZT-RAT|Li	0.200	0.100	700	70.0	
S2	Li|LLZT|LCO	0.038	0.192	300	57.6	Energy & Environmental Science 13, 127 (2020)
	Li|LLZT|Li	0.100	0.050	1000	50.0	
S3	Li|Al-LLZO+PEO|NCM composite	0.030	0.300	100	30.0	J. Industrial and Engineering Chemistry, 71, 445 (2019)
S4	Li–Sn|Ca,Nb-LLZO|Li–Sn	0.050	0.025	100	2.5	Adv. Energy Mater. 8, 1701963 (2017)
S5	Li|LLZT|Li	0.080	0.160	35	5.6	ACS Appl. Mater. Interfaces 8, 10617 (2016)
	Li|LLZT|Li	0.250	0.500	25	12.5	
S6	Li|(Mg coated)Ca,Nb-LLZO|Li	0.100	0.008	225	1.9	Angew. Chem. Int. Ed. 56, 14942 (2017)
S7	Li|Ca,Nb-LLZO|Li	0.100	0.017	141	2.4	Nano Lett. 17, 565 (2017)
S8	Li|LLZT|Li	0.100	0.200	102	20.4	ACS Applied Energy Materials 2, 6720 (2019)
	Li|LLZT|Li	0.200	0.200	104	20.8	
S9	Li|LLZT|Li	0.100	0.008	1260	10.5	Nano Lett. 18, 7414 (2018)
	Li|LLZT|LFP	0.100	0.120	200	24.0	
S10	Li|LLZT-C|Li	0.100	0.100	500	50.0	Journal of the American Chemical Society 140, 6448 (2018)
	Li|LLZT-C|LFP	0.100	0.429	50	21.5	
S11	Li|Graphite coated W-LLZO|Li	0.300	0.300	1000	300.0	ACS Energy Lett. 3, 1212 (2018)
	Li|Graphited coated W-LLZO|NCM523	0.068	0.135	500	67.5	
S12	Li|Gel|LLZO|gel|Li	0.125	0.010	90	0.9	ACS Appl. Mater. Interfaces 9, 18809 (2017)
S13	Li|LLZO|Li	0.200	0.400	100	40.0	Chem. Mater. 29, 7961 (2017)
S14	Li|LLZT|Li	0.100	0.050	1600	80.0	Journal of Materials Chemistry A 7, 14565 (2019)
	Li|LLZT|Li	0.500	0.250	450	112.5	
	Li|LLZT|LFP	0.095	0.516	100	51.6	
	Li|LLZT|NCM523	0.150	0.480	120	57.6	
S15	Li|LLZO|Li	0.200	0.100	90	9.0	Nature Materials 16, 572-579 (2017)

S16	Li+Graphite|LLZT|Li+Graphite	0.300	0.150	250	37.5	Advanced Materials 31, 1807243 (2019)
S17	Li|(Graphite|LLZT|NCM622)Integrated Composite Membrane	0.300	0.300	200	60.0	ACS Appl. Mater. Interfaces 12, 15120 (2020)
S18	Li|Ga-LLZO|Li	0.400	0.200	600	120	Ceramics International, 45, 14991 (2019)
	Li|Ga-LLZO|LFP	0.200	0.600	55	33	
S19	LiMg|LLZO|LiMg	1.000	1.000	250	250.0	Adv. Mater. 31, 1804815 (2019)
	LiMg|LLZO|LiMg	2.000	2.000	250	500.0	
S20	Li|LLZO|Li	1.000	3.000	117	351.0	Journal of Power Sources 396, 314-318 (2018)

Supplementary References

1. Yang, X., Kong, D., Chen, Z., Sun, Y. & Liu, Y. Low-temperature fabrication for transparency Mg doping Li7La3Zr2O12 solid state electrolyte. *Journal of Materials Science: Materials in Electronics* **29**, 1523-1529, doi:10.1007/s10854-017-8062-4 (2018).
2. Ong, S. P. *et al.* Python Materials Genomics (pymatgen): A robust, open-source python library for materials analysis. *Computational Materials Science* **68**, 314-319, doi:https://doi.org/10.1016/j.commatsci.2012.10.028 (2013).
3. Perdew, J. P., Burke, K. & Ernzerhof, M. Generalized Gradient Approximation Made Simple. *Physical Review Letters* **77**, 3865-3868, doi:10.1103/PhysRevLett.77.3865 (1996).
4. Kresse, G. & Furthmüller, J. Efficient iterative schemes for ab initio total-energy calculations using a plane-wave basis set. *Physical Review B* **54**, 11169-11186, doi:10.1103/PhysRevB.54.11169 (1996).
5. Miara, L. J., Richards, W. D., Wang, Y. E. & Ceder, G. First-Principles Studies on Cation Dopants and Electrolyte|Cathode Interphases for Lithium Garnets. *Chemistry of Materials* **27**, 4040-4047, doi:10.1021/acs.chemmater.5b01023 (2015).
6. Richards, W. D., Miara, L. J., Wang, Y., Kim, J. C. & Ceder, G. Interface Stability in Solid-State Batteries. *Chemistry of Materials* **28**, 266-273, doi:10.1021/acs.chemmater.5b04082 (2016).
7. NORDBERG, M. E., MOCHEL, E. L., GARFINKEL, H. M. & OLCOTT, J. S. Strengthening by Ion Exchange. *Journal of the American Ceramic Society* **47**, 215-219, doi:https://doi.org/10.1111/j.1151-2916.1964.tb14399.x (1964).
8. Gy, R. Ion exchange for glass strengthening. *Materials Science and Engineering: B* **149**, 159-165, doi:https://doi.org/10.1016/j.mseb.2007.11.029 (2008).
9. Liu, C. *et al.* Reversible ion exchange and structural stability of garnet-type Nb-doped Li7La3Zr2O12 in water for applications in lithium batteries. *Journal of Power Sources* **282**, 286-293, doi:https://doi.org/10.1016/j.jpowsour.2015.02.050 (2015).
10. Wang, Y. & Lai, W. Phase transition in lithium garnet oxide ionic conductors Li7La3Zr2O12: The role of Ta substitution and H2O/CO2 exposure. *Journal of Power Sources* **275**, 612-620, doi:https://doi.org/10.1016/j.jpowsour.2014.11.062 (2015).
11. Larraz, G., Orera, A. & Sanjuán, M. L. Cubic phases of garnet-type Li7La3Zr2O12: the role of hydration. *Journal of Materials Chemistry A* **1**, 11419-11428, doi:10.1039/C3TA11996C (2013).
12. Galven, C., Fourquet, J.-L., Crosnier-Lopez, M.-P. & Le Berre, F. Instability of the Lithium Garnet Li7La3Sn2O12: Li+/H+ Exchange and Structural Study. *Chemistry of Materials* **23**, 1892-1900, doi:10.1021/cm103595x (2011).
13. Buschmann, H. *et al.* Structure and dynamics of the fast lithium ion conductor "Li7La3Zr2O12". *Physical Chemistry Chemical Physics* **13**, 19378-19392, doi:10.1039/C1CP22108F (2011).
14. Wu, J.-F. *et al.* Gallium-Doped Li7La3Zr2O12 Garnet-Type Electrolytes with High Lithium-Ion

Conductivity. *ACS Applied Materials & Interfaces* **9**, 1542-1552, doi:10.1021/acsami.6b13902 (2017).

15 Samson, A. J., Hofstetter, K., Bag, S. & Thangadurai, V. A bird's-eye view of Li-stuffed garnet-type Li7La3Zr2O12 ceramic electrolytes for advanced all-solid-state Li batteries. *Energy & Environmental Science* **12**, 2957-2975, doi:10.1039/C9EE01548E (2019).

16 Tippens, J. *et al.* Visualizing Chemomechanical Degradation of a Solid-State Battery Electrolyte. *ACS Energy Letters* **4**, 1475-1483, doi:10.1021/acsenergylett.9b00816 (2019).

17 Thompson, T. *et al.* Tetragonal vs. cubic phase stability in Al – free Ta doped Li7La3Zr2O12 (LLZO). *Journal of Materials Chemistry A* **2**, 13431-13436, doi:10.1039/C4TA02099E (2014).

18 Cheng, L. *et al.* The origin of high electrolyte–electrode interfacial resistances in lithium cells containing garnet type solid electrolytes. *Physical Chemistry Chemical Physics* **16**, 18294-18300, doi:10.1039/C4CP02921F (2014).

19 Botros, M. *et al.* Microstrain and electrochemical performance of garnet solid electrolyte integrated in a hybrid battery cell. *RSC Advances* **9**, 31102-31114, doi:10.1039/C9RA07091E (2019).

20 Lin, F. *et al.* Synchrotron X-ray Analytical Techniques for Studying Materials Electrochemistry in Rechargeable Batteries. *Chemical Reviews* **117**, 13123-13186, doi:10.1021/acs.chemrev.7b00007 (2017).

21 Cheng, L. *et al.* Garnet Electrolyte Surface Degradation and Recovery. *ACS Applied Energy Materials* **1**, 7244-7252, doi:10.1021/acsaem.8b01723 (2018).

22 Wiedemann, D. *et al.* Unravelling Ultraslow Lithium-Ion Diffusion in γ-LiAlO2: Experiments with Tracers, Neutrons, and Charge Carriers. *Chemistry of Materials* **28**, 915-924, doi:10.1021/acs.chemmater.5b04608 (2016).

23 Birkl, C. R., Roberts, M. R., McTurk, E., Bruce, P. G. & Howey, D. A. Degradation diagnostics for lithium ion cells. *Journal of Power Sources* **341**, 373-386, doi:https://doi.org/10.1016/j.jpowsour.2016.12.011 (2017).

24 Krewer, U. *et al.* Review—Dynamic Models of Li-Ion Batteries for Diagnosis and Operation: A Review and Perspective. *Journal of The Electrochemical Society* **165**, A3656-A3673, doi:10.1149/2.1061814jes (2018).

25 Watanabe, S., Kinoshita, M., Hosokawa, T., Morigaki, K. & Nakura, K. Capacity fading of LiAlyNi1−x−yCoxO2 cathode for lithium-ion batteries during accelerated calendar and cycle life tests (effect of depth of discharge in charge–discharge cycling on the suppression of the micro-crack generation of LiAlyNi1−x−yCoxO2 particle). *Journal of Power Sources* **260**, 50-56, doi:https://doi.org/10.1016/j.jpowsour.2014.02.103 (2014).

26 Watanabe, S., Kinoshita, M., Hosokawa, T., Morigaki, K. & Nakura, K. Capacity fade of LiAlyNi1−x−yCoxO2 cathode for lithium-ion batteries during accelerated calendar and cycle life tests (surface analysis of LiAlyNi1−x−yCoxO2 cathode after cycle tests in restricted depth of discharge ranges). *Journal of Power Sources* **258**, 210-217, doi:https://doi.org/10.1016/j.jpowsour.2014.02.018 (2014).
